# Medical Sequencing of Candidate Genes for Nonsyndromic Cleft Lip and Palate

**DOI:** 10.1371/journal.pgen.0010064

**Published:** 2005-12-02

**Authors:** Alexandre R Vieira, Joseph R Avila, Sandra Daack-Hirsch, Ecaterina Dragan, Têmis M Félix, Fedik Rahimov, Jill Harrington, Rebecca R Schultz, Yoriko Watanabe, Marla Johnson, Jennifer Fang, Sarah E O'Brien, Iêda M Orioli, Eduardo E Castilla, David R FitzPatrick, Rulang Jiang, Mary L Marazita, Jeffrey C Murray

**Affiliations:** 1 Department of Pediatrics, University of Iowa, Iowa City, Iowa, United States of America; 2 Interdisciplinary Genetics PhD Program, University of Iowa, Iowa City, Iowa, United States of America; 3 Latin American Collaborative Study of Congenital Malformations, Department of Genetics, Federal University of Rio de Janeiro, Rio de Janeiro, Brazil; 4 Latin American Collaborative Study of Congenital Malformations, Department of Genetics, Fiocruz, Rio de Janeiro, Brazil; 5 Center for Medical Education and Investigation, Buenos Aires, Argentina; 6 Cell and Molecular Genetics, Medical Research Council Human Genetics Unit, Edinburgh, United Kingdom; 7 Center for Oral Biology and Department of Biomedical Genetics, University of Rochester, Rochester, New York, United States of America; 8 Center for Craniofacial and Dental Genetics, School of Dental Medicine, University of Pittsburgh, Pittsburgh, Pennsylvania, United States of America; 9 Department of Human Genetics, Graduate School of Public Health, University of Pittsburgh, Pittsburgh, Pennsylvania, United States of America; Western General Hospital, United Kingdom

## Abstract

Nonsyndromic or isolated cleft lip with or without cleft palate (CL/P) occurs in wide geographic distribution with an average birth prevalence of 1/700. We used direct sequencing as an approach to study candidate genes for CL/P. We report here the results of sequencing on 20 candidate genes for clefts in 184 cases with CL/P selected with an emphasis on severity and positive family history. Genes were selected based on expression patterns, animal models, and/or role in known human clefting syndromes. For seven genes with identified coding mutations that are potentially etiologic, we performed linkage disequilibrium studies as well in 501 family triads (affected child/mother/father). The recently reported *MSX1* P147Q mutation was also studied in an additional 1,098 cleft cases. Selected missense mutations were screened in 1,064 controls from unrelated individuals on the Centre d'Étude du Polymorphisme Humain (CEPH) diversity cell line panel. Our aggregate data suggest that point mutations in these candidate genes are likely to contribute to 6% of isolated clefts, particularly those with more severe phenotypes (bilateral cleft of the lip with cleft palate). Additional cases, possibly due to microdeletions or isodisomy, were also detected and may contribute to clefts as well. Sequence analysis alone suggests that point mutations in *FOXE1, GLI2, JAG2, LHX8, MSX1, MSX2, SATB2, SKI, SPRY2,* and *TBX10* may be rare causes of isolated cleft lip with or without cleft palate, and the linkage disequilibrium data support a larger, as yet unspecified, role for variants in or near *MSX2, JAG2,* and *SKI*. This study also illustrates the need to test large numbers of controls to distinguish rare polymorphic variants and prioritize functional studies for rare point mutations.

## Synopsis

Nonsyndromic or isolated cleft lip with or without cleft palate (CL/P) is a birth defect with wide geographic distribution, occurring with an average frequency of 1/700 live births. Treatment can be provided, but it will involve medical, surgical, dental, and psychological personnel. Several different genes have been implicated in different cases. Here the researchers report the results of sequencing 20 different genes in 184 CL/P cases selected with an emphasis on more severe cases and cases with a positive family history for CL/P. Genes were selected based on previous work done by others and by the researchers' group. The authors' results suggest that point mutations in these candidate genes are likely to contribute to about 5% of CL/P, and particularly those with more severe phenotypes (bilateral cleft of the lip with cleft palate). This study also illustrates the need to test large numbers of controls to distinguish rare polymorphic variants and allow investigators to focus functional studies on the rare point mutations that seem to be disease-causing, so that researchers might better understand the mechanisms that play a role in CL/P.

## Introduction

Nonsyndromic or isolated cleft lip with or without palate (CL/P) occurs in wide geographic distribution with an average birth prevalence of 1/700 [1]. CL/P is a complex trait determined by multiple, interacting loci, with additional environmental covariates. Recent work suggests that three to 14 interacting loci provide a good model for genetic effects in CL/P [2].

Studying candidate genes for CL/P selected from animal models and expression patterns is a common strategy [3]. To identify gene(s) involved in CL/P, investigators have used both association and linkage approaches to evaluate genetic contributions. To detect the very small gene effects on CL/P by linkage or linkage disequilibrium strategies, sample sizes need to be large and there needs to be either a common variant in association (for linkage disequilibrium) or a substantial degree of single locus contributions (for linkage). We used direct sequencing as an alternative approach to study candidate genes for CL/P hoping to identify genes with modest effects on the disease. The results of the direct sequencing of *MSX1* [4,5] suggest that point mutations in this gene underlie approximately 2% of CL/P cases. We report here the results of sequencing 20 additional candidate genes for clefts. For seven genes with identified coding mutations that are potentially etiologic, we performed linkage disequilibrium studies as well. For the *MSX1* P147Q mutation reported by Suzuki et al. [5], we investigated an additional 1,098 cleft cases.

## Results

**Figure 1:**
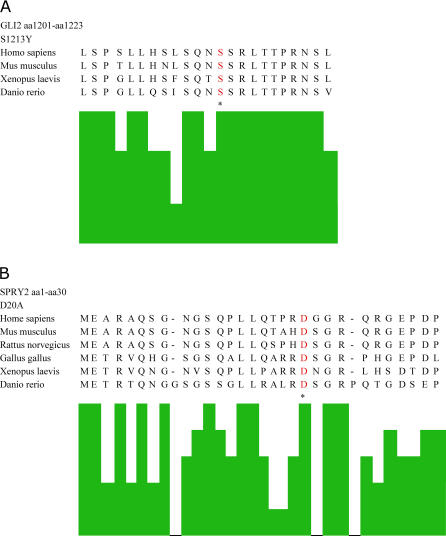
Protein Comparisons of the Available Gene Orthologs for GLI2 S1213Y and SPRY2 D20A GLI2 S1213Y (A) and SPRY2 D20A (B): Green bars indicate degree of conservation in each site. Amino acids in red indicate the mutation sites. All mutation comparisons are available as supplemental material at http://genetics.uiowa.edu/publications.html.

One hundred and forty-nine exons (representing 77,527 nucleotides of DNA sequencing), including exon–intron boundaries and untranslated regions, of 20 genes were screened for mutations in the Iowa and Philippines cleft populations. Table 2 summarizes the number of variants and putative mutations observed. Of the 256 variants seen, 16 missense mutations in nine genes seemed to be of potential etiologic importance. All 16 missense mutations were observed in a single cleft lip and palate case, with the exception of the *SPRY2* D20A and *TBX10* R354Q mutations that were seen in two and three cases respectively. None were seen in the 186 matched controls (Table 3). These mutation sites are not highly conserved across species with the exception of the *SPRY2* and *GLI2* mutations. Both *SPRY2* mutation sites as well as three *GLI2* mutation sites are conserved from *Xenopus* to human (Figure 1; complete data available at http://genetics.uiowa.edu/publication/html). The *JAG2* and the *TBX10* R354Q mutation sites are not conserved in other species orthologs available for study. The sequence surrounding the *JAG2* A657H mutation site is likely a calcium-binding EGF-like domain, which is present in a large number of membrane-bound and extracellular proteins. Also, the *SPRY2* K68N mutation site is in the sprouty domain and inhibits the Ras/mitogen-activated protein kinase (MAPK) cascade, a pathway crucial for developmental processes initiated by activation of various receptor tyrosine kinases.

**Table 1:**
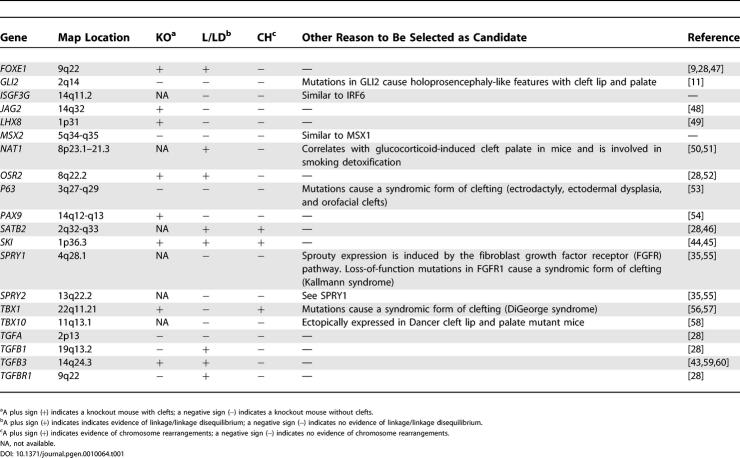
Candidate Genes Studied

All mutations were predicted to be benign by PolyPhen (http://www.bork.embl-heidelberg.de/PolyPhen/) with the exception of the *JAG2* M597I and *SPRY2* D20A that were “possibly damaging” and *GLI2* S1213Y that was “probably damaging” (Table 3). However, with the exception of the *LHX8* E221A, *GLI2* R426Q, and *GLI2* S1213Y mutations, all missense mutations appear to potentially disrupt splicing by either creating or inactivating exonic splicing enhancer sequences (complete information is available as supplemental material at http://genetics.uiowa.edu/publications.html/). None of the mutations identified in this study appear to disrupt possible exonic splicing silencer sequences.

The *SATB2* T190A mutation was not found in the panel of 1064 CEPH controls as well. We also tested the *LHX8* E221A, *SKI* A388V, *SPRY2* D20A, and *TBX10* R354Q mutations in the panel of 1064 CEPH controls after not seeing it in 200 population matched controls. We found the *LHX8* E221A mutation in 17 samples, the *SKI* A388V mutation in nine samples, the *SPRY2* D20A mutation in 60 samples, and the *TBX10* R354Q mutation in six samples. (A complete list is available at our Web site: http://genetics.uiowa.edu/publications.html).

**Figure 2:**
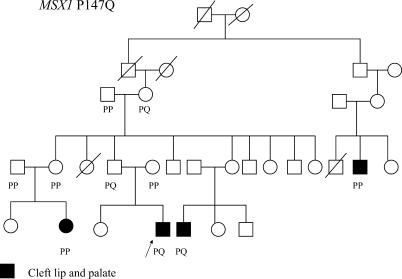
Family Pedigree of the Filipino Family Segregating the MSX1 P147Q Mutation PP (proline–proline) indicates the wild-type genotype. PQ (proline–glutamine) indicates the genotype of the individuals carrying the mutation.

The *MSX1* P147Q mutation was not found in any of 1,671 controls but was found in two Filipino cleft families from a panel of 1,468 cleft cases from the Philippines, which indicates a frequency of 0.14%. The first family has no family history for clefting and the variant segregates from the unaffected mother. The second family has four affected with clefts. The variant was found in two cousins and segregates from the unaffected grandmother to her unaffected son and daughter of a sibship of nine. A first cousin of the proband is affected but does not carry the variant. The last affected, a third cousin once removed, also has a cleft but does not carry the variant (Figure 2).

**Figure 3:**
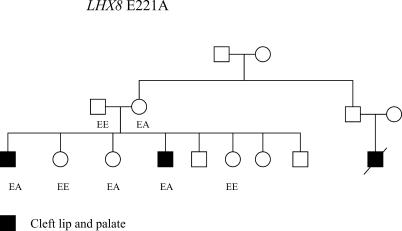
Family Pedigree of the Filipino Proband with the LHX8 E221A Mutation The segregation pattern of the mutation is not consistent with a simple Mendelian model for the disease. EE (glutamic acid–glutamic acid) indicates the wild-type genotype. EA (glutamic acid–alanine) indicates the genotype of the individuals carrying the mutation.

The *LHX8* and *SATB2* mutations were originally seen in single cases from the Philippines. For *SATB2* T190A, the variant was transmitted from the unaffected mother. The *LHX8* E221A mutation was also transmitted from the unaffected mother and is present in one affected and one unaffected person but was absent in two unaffected siblings from a sibship of eight brothers and sisters (Figure 3). Both mutation sites are conserved between human and mouse (see Appendix at http://genetics.uiowa.edu/publications.html/). The *SKI* A388V mutation was seen in a case from Iowa. The mutation was transmitted from the mother and the mutation site is conserved between humans and species of fish and frog. Two cases from Iowa presented with the *SPRY2* D20A mutation. Of these, one had parental DNA samples available and the mutation segregated from the unaffected mother. Three cleft individuals from Iowa presented the *TBX10* R354Q mutation. In the two cases with parent samples available, one received the mutated allele from the mother and the other from the father (both unaffected).

Two additional interesting observations were made. An Iowa case presented with isolated cleft lip with cleft palate, and no family history of clefts or any features of DiGeorge syndrome was found to be homozygous for an intronic variant in the *TBX1* gene (189 nucleotides into intron 8). This variant was not present in 186 matched controls. We tested this case for the presence of two copies of the ubiquitin fusion degradation gene *(UFD1L)*, using an assay for DiGeorge or 22q syndrome [6], and the results were normal. Parental samples are not available to further study this case, nor is there enough DNA available to confirm a possible deletion by Southern blot analysis. The finding of a single rare homozygote variant suggests the possibility of a microdeletion or segmental isodisomy of this region. We tested four microsatellite markers (D22S420, D22S1685, D22S683, and D22S445) on both proband and mother samples. The mother presents distinct genotypes from the proband for D22S420 and D22S683 markers, however this finding does not exclude a segmental maternal isodisomy because the interval between these two markers, which contains *TBX1,* is 32 cM (data not shown).

The second observation involved four Filipino cases that presented a missense mutation in the last *SKI* amino acid (P728L). This mutation was not found in 186 matched controls. One case appears to have a de novo *SKI* P728L mutation. This case presented with an isolated right cleft lip and cleft palate and positive family history for clefts. Neither parent had this variant and their biological relationship to the case was confirmed after testing twenty polymorphic markers. Of the other three cases, one had a positive family history for clefts. For this case, we tested the parents, the paternal grandparents and three siblings out of a 15 sibship progeny. One of the tested siblings is affected with a right cleft lip and cleft palate associated with microcephaly. The *SKI* P728L variant segregated from the unaffected grandfather to the unaffected father. Of the three siblings tested, the two unaffected siblings had the variant, but the affected sibling did not. Based on this family, we concluded that this *SKI* P728L variant is probably not an etiologic mutation and included it in the column of non-synonymous coding variants in Table 2.

**Table 2:**
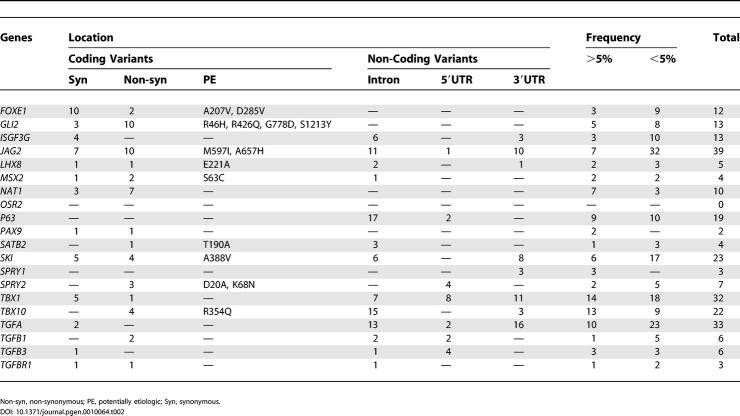
Summary of Variants Found by Direct Sequence

**Table 3:**
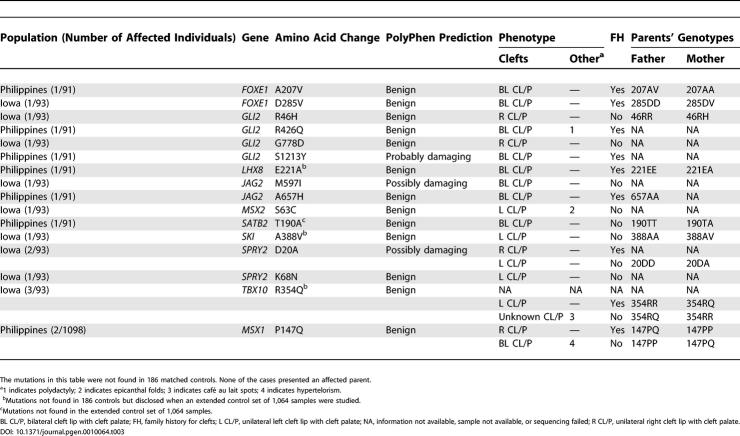
Potential Mutations Found in the Present Study

Linkage disequilibrium studies were performed for the genes *GLI2, JAG2, MSX2, SATB2, SKI, SPRY2,* and *TBX10* in which likely etiologic missense mutations had been observed (complete information is available as supplemental material at http://genetics.uiowa.edu/publications.html/; results for *FOXE1* were previously reported in Marazita et al. [7]). No single nucleotide polymorphism tested showed evidence for deviation from Hardy-Weinberg equilibrium in either the affected or unaffected individuals (data not shown). The haplotype analysis using the HBAT function of FBAT (http://www.biostat.harvard.edu/~fbat/fbat.htm) demonstrated borderline associations between *MSX2* in both Filipino (*p* = 0.001) and Iowa (*p* = 0.008) populations, between *JAG2* and the Filipinos (*p* = 0.004), and between both *SATB2* (*p* = 0.03) and *TBX10* (*p* = 0.04) and the Iowa population. However, when we combined the *MSX2* haplotype data for both Filipino and Iowa populations, the association was weaker (*p* = 0.09). We also observed an association between CL/P and snp2 (rs2843159) in *SKI* (*p* = 0.000004) in the Filipino population. This association between CL/P and *SKI* in Filipinos was also suggested by the haplotype analysis (*p* = 0.0002). In addition, the same *SKI* snp2 marker showed association with cleft lip only in the South American clefting population from the Latin American Collaborative Study of Congenital Malformations (ECLAMC) (*p* = 0.004).

## Discussion

Point mutations in the candidate genes *FOXE1, GLI2, MSX2, SKI, SATB2,* and *SPRY2* appear in aggregate to contribute to as much as 6% of isolated cleft lip and palate cases, enriched for cases with bilateral cleft of the lip with cleft palate and a positive family history. The mutations found in this study are conserved in other mammals, may disrupt exonic splicing enhancer sequences, and were not found in between 400 to 2,000 control chromosomes. The *JAG2* M597I and A657H mutations, although they appear to disrupt exonic splicing enhancer sequences and possibly damage the JAG2 protein, according to the PolyPhen prediction, are not conserved in other species and may be rare polymorphic sites.

Testing a larger number of control samples proved to be a useful way to differentiate rare polymorphisms from etiologic mutations. The *LHX8* E221A, *SATB2* T190A, *SKI* A388V, *SPRY2* D20A, and *TBX10* R354Q variants initially were not observed in approximately 200 matched controls. This number of controls is commonly used to assume that if a variant is not present, it is likely causal despite models that have shown that a larger number of controls is useful in eliminating rare variants [8]. However, when we tested these variants in the extended set of 1,064 controls, we found the *LHX8* E221A variant in 17 individuals, the *SKI* A388V mutation in nine, the *SPRY2* D20A mutation in 60 individuals, and the *TBX10* R354Q in six individuals. Although the presence of the amino acid changes in unaffected controls does not exclude them from playing a role in CL/P, it does place them in a lower priority group for additional functional analysis and makes them difficult to use in any applied genetic counseling setting as they may be, at best, modifiers with low penetrance contributory alleles.

Some variants, such as the *SKI* A388V mutation, which is conserved back to *Xenopus* and *Tilapia* but is found in controls, demonstrate that species conservation alone maybe not enough to argue for an etiologic role of a given variant.

Our study illustrates the difficulties of defining as causal a mutation rarely seen in the population. Many of the missense mutations found in the cases studied were seen only when we extended our screen to a larger control group comprised of samples from ethnically diverse groups from almost all parts of the world. In addition, none of the mutations segregated from an affected parent. Incomplete penetrance is likely the explanation for the mutations that may be causal as has been clearly shown for other genes that contribute to clefting such as *MSX1* [9] and *FGFR1* [10] mutations. It is likely that we found these mutations only because we tested cases more likely to present a stronger genetic contribution (cases with positive family history and bilateral cleft of the lip with cleft palate). Mutations like the *MSX1* P147Q and others that appear to show incomplete penetrance are comparable to autosomal dominant disorders resulting from mutations in *SCN5A* [11], *IRF6* [12], or *NKX2.5* [13].

The *MSX1* P147Q mutation was seen in two cleft cases from the Philippines, but in none of the over 1,600 controls. It appears that this specific mutation underlies approximately 0.15% cases of apparent isolated CL/P. As shown previously this variant results in variable expression and decreased penetrance that make prospective studies of its phenotypic outcome necessary before accurate genetic counseling risk can be measured [5].

Rigorous demonstration that a mutation disrupts a genuine exonic splicing enhancer requires that the sequence autonomously promote splicing and that enhancement be absent in the mutant. An advantage of the score matrix approach [14] is that it allows direct testing of predicted effects on individual putative enhancer sites, rather than having to characterize exonic splicing enhancers by testing multiple random mutations and/or deletions along an exon. All 15 mutations on Table S2 appear to inactivate and/or create a predicted exonic splicing enhancer of at least one of the four serine/arginine-rich (SR) proteins. However, the presence of a high-score motif in a sequence does not necessarily identify that sequence as an exonic splicing enhancer in its native context.

In *TBX1,* we found one rare intronic variant in homozygous form in an Iowa cleft case, which could indicate that clefts arise from recessive functional intronic mutations in *TBX1,* or microdeletions that cannot be visualized by direct sequencing. This case does not have a 22q deletion involving the *UFD1L* gene. Detecting this rare homozygote in the absence of this variant in any other of the 400 people tested suggests these individuals may be identical by descent at this locus and gene. This variant itself, or others in linkage disequilibrium in *TBX1,* might be a hypomorphic allele whose joint presence results in enough change in gene expression or function to trigger a phenotype. Therefore, other alleles in regulatory regions of *TBX1* should be a priority for identification.

Previous work from our group has screened *FGFR1, IRF6, MSX1, TGFA,* and *TGFB3* for mutations on cleft cases. Point mutations in *MSX1* appear to contribute approximately to 2% of all CL/P cases [4]. *FGFR1* point mutations also appear to contribute to CL/P. In addition, *FGFR1* loss-of-function mutations can cause forms of Kallmann syndrome that mimic isolated CL/P at birth and during childhood. Mutations in *IRF6,* which cause the syndromic forms of clefts, the Van der Woude and popliteal pterygium syndromes [12], were not found in the same collection of cleft cases as the present study, although rare non-coding variants in conserved regions were disclosed. However, *IRF6* is strongly associated with CL/P, and it is likely a genetic modifier for clefts [15,16]. Previously for *TGFA,* five variants in conserved non-coding segments were found in individual cases but not seen in 278 controls [17]. In the present study we found another nine non-coding rare variants in single individuals, but we did not find the original five reported rare variants or any coding mutation that could be etiologic. If these variants are disease-causing mutations, they could explain, not only the conflicting results from association studies of isolated orofacial clefts and *TGFA* variants [18], but also the linkage studies that suggest a cleft susceptibility loci in 2p13, the *TGFA* locus [7]. For *TGFB3,* we previously reported one missense mutation (K130R) in a cleft palate–only case not seen in 350 controls [19]. We did not find this or any other mutations in *TGFB3* in our current study population.

Loss-of-function mutations in *GLI2* are associated with pituitary anomalies and holoprosencephaly-like features [20]. In this report, the three pedigrees segregating *GLI2* loss-of-function mutations with complete clinical information presented orofacial clefts and polydactyly. We found four missense mutations in *GLI2* in highly conserved amino acids. One of the cases also presented with polydactyly (Table 3).

We performed linkage disequilibrium studies in the genes that we found potentially disease-causing missense mutations. We found association between a marker (rs2843159) and a haplotype in *SKI* in the Filipino population. This association was also found in an independent population dataset from South America. The *SKI* locus, 1p36.3, was previously suggested as a cleft susceptibility loci in Caucasians [21,22]. *Ski* null mice present with clefts involving the lip [22], and the association we found appears to be stronger when cases with the involvement of the lip are included.

The possible trend for an association between clefts with a palate phenotype and *SATB2* are in agreement with the cytogenetic evidence. Deletions and balanced translocations point to the existence of a locus on 2q32-q33, for which haploinsufficiency results in isolated cleft palate. A mutation analysis of *SATB2* (located at 2q32-q33) in 70 unrelated patients with isolated cleft palate only did not reveal any coding region variants [23]. However, a meta-analysis of 13 genome scans for clefts indicated 2q32-q35 as a clefting susceptibility locus [7]. We studied 184 cleft lip and palate cases and found one missense mutation in *SATB2* (T190A) that was not seen in approximately 1,200 controls. Based on the linkage disequilibrium and mutation analysis results of our study, we believe a regulatory element outside *SATB2* coding regions may be implicated in clefting.

In summary, point mutations in six of the 20 candidate genes selected from expression, animal, and human data may to contribute to about 5% of isolated clefts, more likely those with more-severe phenotypes and/or a positive family history. Etiologic variants in regulatory elements of *SKI, JAG2,* and *MSX2* may contribute to isolated clefts as well. Predictions by ESEfinder (http://rulai.cshl.edu/tools/ESE/) and PolyPhen regarding the function of the missense mutations found in this study, as well as exonic splicing enhancement and protein damaging, are challenging to interpret.

A major challenge in these studies was the frequent absence of a cleft phenotype in near relatives of an affected proband with a cleft and a rare missense mutation. In some cases these variants may not be etiologic, but in others, reduced penetrance for the cleft may be an active force as has been seen commonly in clefts [9,10] and other birth defects such as congenital hearth disease. Similarly these mutations may only be modifiers of the phenotype.

Cases due to microdeletions or isodisomy may contribute to clefts as well. This study illustrates the validity of testing greater numbers of controls to determine rare polymorphic variants and prioritize functional studies for rare point mutations. Given other recent data on the roles of *FGFR1, IRF6,* and *MSX1* in isolated CL/P, one can begin to consider sequencing of a panel of high-probability candidate genes for genetic counseling indication. Although issues of penetrance and even etiology for any given mutation are not yet resolved, progress in this direction is now measurable.

## Materials and Methods

Two collections of CL/P cases, 91 from the Philippines and 93 from Iowa, United States, were used to search for mutations in 20 candidate genes (Table 1). We selected the more-severe cases from those available to us, and the sequenced samples were enriched by bilateral cleft lip and palate cases with a positive family history for clefts (39/91 from the Philippines and 16/93 from Iowa). Two cases were later found to have associated features—one with Stickler syndrome in which family history was initially not available, and a second with polydactyly. Cases from the Philippines were studied under the auspices of Operation Smile International [24]. Patients were seen and examined by a board certified clinical geneticist (JCM or colleagues; see Acknowledgments) at one of four sites within the Philippines (Cavite, Kalibo, Cebu, and Negros). Iowa cases were collected through the Iowa Birth Defects Registry [25]. Signed consents were obtained from all participants before a blood sample was obtained. DNA was extracted according to published protocols.

For each of the 20 candidate genes, all exons and 5′ and 3′ untranslated regions were sequenced in both directions. Primer sequences and PCR conditions are available on our Web site (http://genetics.uiowa.edu/publications.html). Primers for *FOXE1, GLI2, MSX2, OSR2,* and *TGFBR1* were obtained from the literature [20,26–29]. Cycle sequencing was performed in a 20-μl reaction using 4 μl of Applied Biosystems Big Dye Terminator sequencing reagent, 1 μl of 5 μM sequencing primer, 1 μl of DMSO, 4 μl of 2.5× Buffer, and 2.5 ng/100 base pair of DNA template. Following a denaturation step at 96 °C for 30 s, reactions were cycle sequenced at 96 °C for 10 s, 50 °C for 5 s, and 60 °C for 4 min for 40 cycles. Cleanup was performed using standard protocols. Samples were resuspended in 40 to 100 μl of ddH_2_O, and 2.5 μl were then injected on an Applied Biosystems 3700 sequencer. The Applied Biosystems sequence software (version 2.1.2) was used for lane tracking and first pass base calling. Chromatograms were transferred to a Unix workstation, base called with PHRED (version 0.961028), assembled with PHRAP (version 0.960731), scanned by POLYPHRED (version 0.970312), and the results viewed with CONSED (version 4.0) [30]. When the results indicated a possible new variant, the case sample was resequenced, as well as other available family members, and population controls. These data were analyzed using the same method.

For any coding variant, we performed direct sequencing in 186 population-matched controls. Control populations were collected as described above for the cases and consisted of individuals with no CL/P or other recognized birth defect from adults at the same sites where cases were collected. If the variant was found in one or more controls, it was considered a polymorphism. To expand the number of controls tested, we developed allele-specific assays for the *LHX8* E221A, *SATB2* T190A, *SKI* A388V, *SPRY2* D20A, and *TBX10* R354Q mutations. We tested them in the CEPH Diversity Cell Line Panel [31], which is comprised of 1,064 DNA samples from cultured lymphoblastoid cell lines derived from individuals representing 51 different human populations.

We also developed an assay for the *MSX1* P147Q missense mutation, recently reported in three Vietnamese cleft families [5]. Besides the 1,064 CEPH Diversity Cell Line Panel controls, we tested the *MSX1* P147Q assay in an additional 607 Filipino controls and in a collection of 1,468 cleft cases from the Philippines as well.

For the nine genes with potentially etiologic missense mutations, we identified orthologs through BLAST search of the non-redundant database using *Homo sapiens FOXE1, GLI2, LHX8, JAG2, MSX2, SATB2, SKI, SPRY2,* and *TBX10,* as reference sequences. We performed protein sequence comparisons with the available species. We also used the ESEfinder software available online to predict the presence of exonic splicing enhancers [14], which appear to be prevalent, and may be present in most, if not all exons [32,33]. We screened the 141 exonic splicing silencer decamers that were identified by Wang et al. [34] to check if any of those could be affected by the missense mutations we found. Finally, we used the PolyPhen software, also available online, to predict the impact of the amino acid substitutions identified on the structure and function of the human protein [35–37].

Two single nucleotide polymorphisms in weak linkage disequilibrium with each other were selected for each population to perform linkage disequilibrium studies in the genes with missense mutations in cases but not in controls. Four single nucleotide polymorphisms were chosen for *GLI2* based on the International HapMap Project's linkage disequilibrium pattern of the gene (data not shown). Frequency of the alleles can be found in the supplemental material (http://genetics.uiowa.edu/publications.html). TaqMan-based assays [38] were performed on Applied Biosystems 7900 HT Sequence Detection System (Applied Biosystems, Foster City, California, United States). For one marker in *SKI* (rs2843159), we used a kinetic polymerase chain reaction assay previously reported [39]. These linkage disequilibrium studies were composed of 296 complete triads (mother/father/affected child) from the Philippines and 205 from Iowa. These samples were obtained as described above for cases and controls investigated by sequencing. The Family Based Association Test (FBAT) [40–42] program was used for this analysis. Significance figures were accounted for using Bonferroni correction taking into account the number of tests carried out [43]. With the Bonferroni correction, alpha is 0.0003 (0.05/192 comparisons) for the individual marker analysis and 0.0001 (0.05/384 comparisons) for the haplotype analysis. Linkage disequilibrium studies for *FOXE1* were previously reported in Marazita et al. [7].

A third clefting population sample set of 434 case/mother pairs from South America was used to replicate any significant association. These population samples are derived from ECLAMC, which is a hospital-based birth defects registry study that includes sites in Argentina, Bolivia, Brazil, Chile, Ecuador, Paraguay, Uruguay, and Venezuela. This study population has previously been described in detail [44,45]. To analyze the ECLAMC samples, the likelihood ratio test (LRT) of Weinberg [46] was applied under the assumption that the distribution of paternal alleles is the same as maternal.

## Supporting Information

Figure S1 Protein Comparisons of the Available Gene Orthologs for the Mutations Found in the Present Study Green bars indicate degree of conservation in each site. Amino acids in red indicate the mutation sites. All mutation comparisons are available as supplemental material at http://genetics.uiowa.edu/publications.html.
(56 KB PDF)

Table S1 Primers and PCR Conditions (51 KB PDF)

Table S2 Exonic Splicing Enhancer Prediction Analysis for the Missense Mutations Found in the Present Study. Nucleotides in red indicate the mutation sites. (61 KB PDF)

Table S3 Mutations Screened in the CEPH Diversity Cell Line Panel (48 KB PDF)

Table S4 Markers Selected for Linkage Disequilibrium Studies (49 KB PDF)

Table S5 Haplotype Frequencies (46 KB PDF)

Table S6 Linkage Disequilibrium Studies of Candidate Genes for Clefting (50 KB PDF)

Table S7 Haplotype Analysis (54 KB PDF)

Table S8 SKI LRT Results for the South American (ECLAMC) Clefting Samples (40 KB PDF)

### Accession Numbers

The National Center for Biotechnology Information (http://www.ncbi.nlm.nih.gov/) Unigene accession numbers for the genes discussed in this paper are *Homo sapiens FOXE1* (NM_004473), *GLI2* (NP_084655), *JAG2* (NM_002226), *LHX8* (AY449521), *MSX2* (NM_002449), *SATB2* (NM_015265), *SKI* (AY334556), *SPRY2* (NM_ 005842), and *TBX10* (AY229977).

## Abbreviations

CEPH: Centre d'Étude du Polymorphisme Humain
CL/P: cleft lip with or without cleft palate
ECLAMC: Latin American Collaborative Study of Congenital Malformations
LRT: likelihood ratio test

## References

[pgen-0010064-b001] Mossey PA, Little J, Wyszynski DF (2002). Epidemiology of oral clefts: An international perspective. Cleft lip and palate. From origin to treatment.

[pgen-0010064-b002] Schliekelman P, Slatkin M (2002). Multiplex relative risk and estimation of the number of loci underlying an inherited disease. Am J Hum Genet.

[pgen-0010064-b003] Murray JC, Schutte BC (2004). Cleft palate: Players, pathways, and pursuits. J Clin Invest.

[pgen-0010064-b004] Jezewski PA, Vieira AR, Nishimura C, Ludwig B, Johnson M (2003). Complete sequencing shows a role for MSX1 in non-syndromic cleft lip and palate. J Med Genet.

[pgen-0010064-b005] Suzuki Y, Jezewski PA, Machida J, Watanabe Y, Shi M (2004). In a Vietnamese population, MSX1 variants contribute to cleft lip and palate. Genet Med.

[pgen-0010064-b006] Kariyazono H, Ohno T, Ihara K, Igarashi H, Joh-o K (2001). Rapid detection of the 22q11.2 deletion with quantitative real-time PCR. Mol Cellular Probes.

[pgen-0010064-b007] Marazita ML, Murray JC, Lidral AC, Arcos-Burgos M, Cooper ME (2004). Meta-analysis of 13 genome scans reveals multiple cleft lip/palate genes with novel loci on 9q21 and 2p32–35. Am J Hum Genet.

[pgen-0010064-b008] Collins JS, Schwartz CE (2002). Detecting polymorphisms and mutations in candidate genes. Am J Hum Genet.

[pgen-0010064-b009] van den Boogard MJH, Dorland M, Beemer FA, van Amstel HKP (2000). MSX1 mutation is associated with orofacial clefting and tooth agenesis in humans. Nat Genet.

[pgen-0010064-b010] Dodé C, Levilliers J, Dupont JM, De Paepe A, Le Dû N (2003). Loss-of-function mutations in FGFR1 cause autosomal dominant Kallmann syndrome. Nat Genet.

[pgen-0010064-b011] Hong K, Brugada J, Oliva A, Berruezo-Sanchez A, Potenza D (2004). Value of electrocardiographic parameters and ajmaline test in the diagnosis of Brugada syndrome caused by SCN5A mutations. Circulation.

[pgen-0010064-b012] Kondo S, Schutte BC, Richardson RJ, Bjork BC, Knight AS (2002). Mutations in interferon regulatory factor 6 cause Van der Woude and popliteal pterygium syndromes. Nat Genet.

[pgen-0010064-b013] Watanabe Y, Benson DW, Yano S, Akagi T, Yoshino M (2002). Two novel frameshift mutations in NKX2.5 result in novel features including visceral inversus and sinus venosus type ASD. J Med Genet.

[pgen-0010064-b014] Cartegni L, Wang J, Zhu Z, Zhang MQ, Krainer AR (2003). ESEfinder: A web resource to identify exonic splicing enhancers. Nucleic Acid Res.

[pgen-0010064-b015] Zucchero T, Cooper ME, Maher BS, Daack-Hirsch S, Nepomuceno B (2004). Interferon regulatory factor (IRF6) is a modifier for isolated cleft lip and palate. New Engl J Med.

[pgen-0010064-b016] Scapoli L, Palmieri A, Martinelli M, Pezetti F, Carinci P (2005). Strong evidence of linkage disequilibrium between polymorphisms at the IRF6 locus and nonsyndromic cleft lip with or without cleft palate, in an Italian population. Am J Hum Genet.

[pgen-0010064-b017] Machida J, Yoshiura K, Funkhauser CD, Natsume N, Kawai T (1999). Transforming growth factor-α (TGFA): Genomic structure, boundary sequences, and mutation analysis in nonsyndromic cleft lip/palate and cleft palate only. Genomics.

[pgen-0010064-b018] Mitchell LE (1997). Transforming growth factor α locus and nonsyndromic cleft lip with or without cleft palate: A reappraisal. Genet Epidemiol.

[pgen-0010064-b019] Lidral AC, Romitti PA, Basart AM, Doetschman T, Leysens NJ (1998). Association of MSX1 and TGFB3 with nonsyndromic clefting in humans. Am J Hum Genet.

[pgen-0010064-b020] Roessler E, Du YZ, Mullor JL, Casas E, Allen WP (2003). Loss-of-function mutations in the human GLI2 gene are associated with pituitary anomalies and holoprosencephaly-like features. Proc Natl Acad Sci U S A.

[pgen-0010064-b021] Prescott NJ, Lees MM, Winter RM, Malcolm S (2000). Identification of susceptibility loci for nonsyndromic cleft lip with or without cleft palate in a two stage genome scan of affected sib-pairs. Hum Genet.

[pgen-0010064-b022] Colmenares C, Heilstedt HA, Shaffer LG, Schwartz S, Berk M (2002). Loss of the SKI proto-oncogene in individuals affected with 1p36 deletion syndrome is predicted by strain-dependent defects in Ski^−/−^ mice. Nat Genet.

[pgen-0010064-b023] FitzPatrick DR, Carr IM, McLaren L, Leek JP, Wightman P (2003). Identification of SATB2 as the cleft palate gene on 2q32-q33. Hum Mol Genet.

[pgen-0010064-b024] Murray JC, Daack-Hirsch S, Buetow KH, Munger R, Espina L (1997). Clinical and epidemiologic studies of cleft lip and palate in the Philippines. Cleft Palate Craniofac J.

[pgen-0010064-b025] Romitti PA, Munger RG, Murray JC, Daack-Hirsch S, Hanson JW (1998). The effect of follow-up on limiting participation bias in genetic epidemiologic investigations. Eur J Epidemiol.

[pgen-0010064-b026] Wilkie AOM, Tang Z, Elanko N, Walsh S, Twigg SRF (2000). Functional haploinsufficiency of the human homeobox gene MSX2 causes defects in skull ossification. Nat Genet.

[pgen-0010064-b027] Castanet M, Park SM, Smith A, Bost M, Léger J (2002). A novel loss-of-function mutation in TTF-2 is associated with congenital hypothyroidsm, thyroid agenesis and cleft palate. Hum Mol Genet.

[pgen-0010064-b028] Debeer P, de Ravel TJL, Devriendt K, Fryns JP, Huysmans C (2002). Human homologues of Osr1 and Osr2 are not involved in a syndrome with distal limb deficiencies, oral abnormalities, and renal defects. Am J Hum Genet.

[pgen-0010064-b029] Zhang HT, Fei QY, Chen F, Qi QY, Zou W (2003). Mutational analysis of the transforming growth factor β receptor type I gene in primary non-small cell lung cancer. Lung Cancer.

[pgen-0010064-b030] Nickerson DA, Tobe VO, Taylor SL (1997). PolyPhred: Automating the detection and genotyping of single nucleotide substitutions using fluorescence-based resequencing. Nucleic Acids Res.

[pgen-0010064-b031] Cann HM, Toma C, Cazes L, Legrand MF, Morel V (2002). A human diversity cell line panel. Science.

[pgen-0010064-b032] Blencowe BJ (2000). Exonic splicing enhancers: Mechanism of action, diversity and role in human genetic diseases. Trends Biochem Sci.

[pgen-0010064-b033] Graveley BR (2000). Sorting out the complexity of SR protein functions. RNA.

[pgen-0010064-b034] Wang Z, Rolish ME, Yeo G, Tung V, Mawson M (2004). Systematic identification and analysis of exonic splicing silencers. Cell.

[pgen-0010064-b035] Sunyaev S, Ramensky V, Bork P (2000). Towards a structural basis of human non-synonymous single nucleotide polymorphisms. Trends Genet.

[pgen-0010064-b036] Sunyaev S, Ramensky V, Koch I, Lathe W, Kondrashov AS (2001). Prediction of deleterious human alleles. Hum Mol Genet.

[pgen-0010064-b037] Ramesnky V, Bork P, Sunyaev S (2002). Human non-synonymous SNPs: Server and survey. Nucleic Acids Res.

[pgen-0010064-b038] Ranade K, Chang MS, Ting CT, Pei D, Hsiao CF (2001). High-throughput genotyping with single nucleotide polymorphisms. Genome Res.

[pgen-0010064-b039] Shi M, Caprau D, Dagle J, Christiansen L, Christensen K (2004). Application of kinetic polymarase chain reaction and molecular beacon assays to pooled analyses and high-throughput genotyping for candidate genes. Birth Defects Res A Clin Mol Teratol.

[pgen-0010064-b040] Laird NM, Horvath S, Xu X (2000). Implementing a unified approach to family-based tests of association. Genet Epidemiol 19 Suppl.

[pgen-0010064-b041] Rabinowitz D, Laird N (2000). A unified approach to adjusting association tests for population admixture with arbitrary pedigree structure and arbitrary missing marker information. Hum Hered.

[pgen-0010064-b042] Horvath S, Xu X, Laird NM (2001). The family based association test method: strategies for studying general genotype-phenotype associations. Eur J Hum Genet.

[pgen-0010064-b043] Bonferroni CE (1936). Teoria statistica delle classi e calcolo delle probabilità. Pubblicazioni del R Istituto Superiore di Scienze Economiche e Commerciali di Firenze.

[pgen-0010064-b044] Vieira AR, Karras JC, Orioli IM, Castilla EE, Murray JC (2002). Genetic origins in a South American clefting population. Clin Genet.

[pgen-0010064-b045] Vieira AR, Orioli IM, Castilla EE, Cooper ME, Marazita ML (2003). MSX1 and TGFB3 contribute to clefting in South America. J Dent Res.

[pgen-0010064-b046] Weinberg CR (1999). Allowing for missing parents in genetic studies of case-parent triads. Am J Hum Genet.

[pgen-0010064-b047] De Felice M, Ovitt C, Biffali E, Rodriguez-Mallon A, Arra C (1998). A mouse model for hereditary thyroid dysgenesis and cleft palate. Nat Genet.

[pgen-0010064-b048] Jiang R, Lan Y, Chapman HD, Shawber C, Norton CR (1998). Defects in limb, craniofacial, and thymic development in Jagged2 mutant mice. Genes Dev.

[pgen-0010064-b049] Zhao Y, Guo YJ, Tomac AC, Taylor NR, Grinberg A (1999). Isolated cleft palate in mice with a targeted mutation of the LIM homeobox gene Lhx8. Proc Natl Acad Sci U S A.

[pgen-0010064-b050] Karolyi J, Erickson RP, Liu S, Killewald L (1990). Major effect on teratogen-induced facial clefting in mice determined by a single genetic region. Genetics.

[pgen-0010064-b051] Lammer EJ, Shaw GM, Iovannisci DM, van Waes J, Finnell RH (2004). Maternal smoking and the risk of orofacial clefts: Susceptibility with NAT1 and NAT2 polymorphisms. Epidemiology.

[pgen-0010064-b052] Lan Y, Ovitt CE, Cho ES, Maltby KM, Wang Q (2004). Odd-skipped related 2 (Osr2) encodes a key intrinsic regulator of secondary palate growth and morphogenesis. Development.

[pgen-0010064-b053] Celli J, Duijf P, Hamel BCJ, Bamshad M, Kramer B (1999). Heterozygous germline mutations in the p53 homolog p63 are the cause of EEC syndrome. Cell.

[pgen-0010064-b054] Peters H, Neubuser A, Kratochwil K, Balling R (1998). Pax9-deficient mice lack pharyngeal pouch derivatives and teeth and exhibit craniofacial and limb abnormalities. Genes Dev.

[pgen-0010064-b055] Reich A, Sapir A, Shilo B (1999). Sprouty is a general inhibitor of receptor tyrosine kinase signaling. Development.

[pgen-0010064-b056] Jerome LA, Papaioannou VE (2001). DiGeorge syndrome phenotype in mice mutant for the T-box gene, Tbx1. Nat Genet.

[pgen-0010064-b057] Yagi H, Furutani Y, Hamada H, Sasaki T, Asakawa S (2003). Role of TBX1 in human del22q11.2 syndrome. Lancet.

[pgen-0010064-b058] Bush JO, Lan Y, Jiang R (2004). The cleft lip and palate defects in Dancer mutant mice result from gain of function of the Tbx10 gene. Proc Natl Acad Sci U S A.

[pgen-0010064-b059] Kaartinen V, Voncken JW, Shuler C, Warburton D, Bu D (1995). Abnormal lung development and cleft palate in mice lacking TGF-beta-3 indicates defects of epithelial-mesenchymal interaction. Nat Genet.

[pgen-0010064-b060] Proetzel G, Pawlowski SA, Wiles MV, Yin M, Boivin GP (1995). Transforming growth factor-B3 is required for secondary palate fusion. Nat Genet.

