# Supplementary material for: Medical Sequencing of Candidate Genes for Nonsyndromic Cleft Lip and Palate
Source: PLoS Genet. 2005 Dec 2;1(6):e64. doi: 10.1371/journal.pgen.0010064 (PMC1298935; doi:10.1371/journal.pgen.0010064)
Supplement: Supplementary file 1 [file pgen.0010064.sg001.pdf]

FOXE1 aa187-aa210
A207V

Homo sapiens	A A R P P Y P G A V Y A G Y A P P S L A A P P P
Mus musculus	A A R P A Y P G A V Y A G Y A P P - L A A P P P
Rattus norvegicus	A A R P A Y P G A V Y A G Y A P P - L A A P P P
	*

FOXE1 aa271-aa288
D285V

Homo sapiens	P A N P S A Y A A A Y A G P D G A Y
Mus musculus	P V N P A A Y A A A Y A G P D G A Y
Rattus norvegicus	P V N P A A Y A A A Y A G P D G A Y
	*

GLI2 aa41-aa50
R46H

Homo sapiens	V A I H K R S K V K T E P E G L R P A S P L A L
Mus musculus	I T I H K R S K V K T E A E G L R P A S P L G L
Xenopus laevis	Q V I H K R N K V K T E E E V L Q R D S P S P P
Danio rerio	P M I F K R S K V K T E A E G P H P I S P G S Q
	*

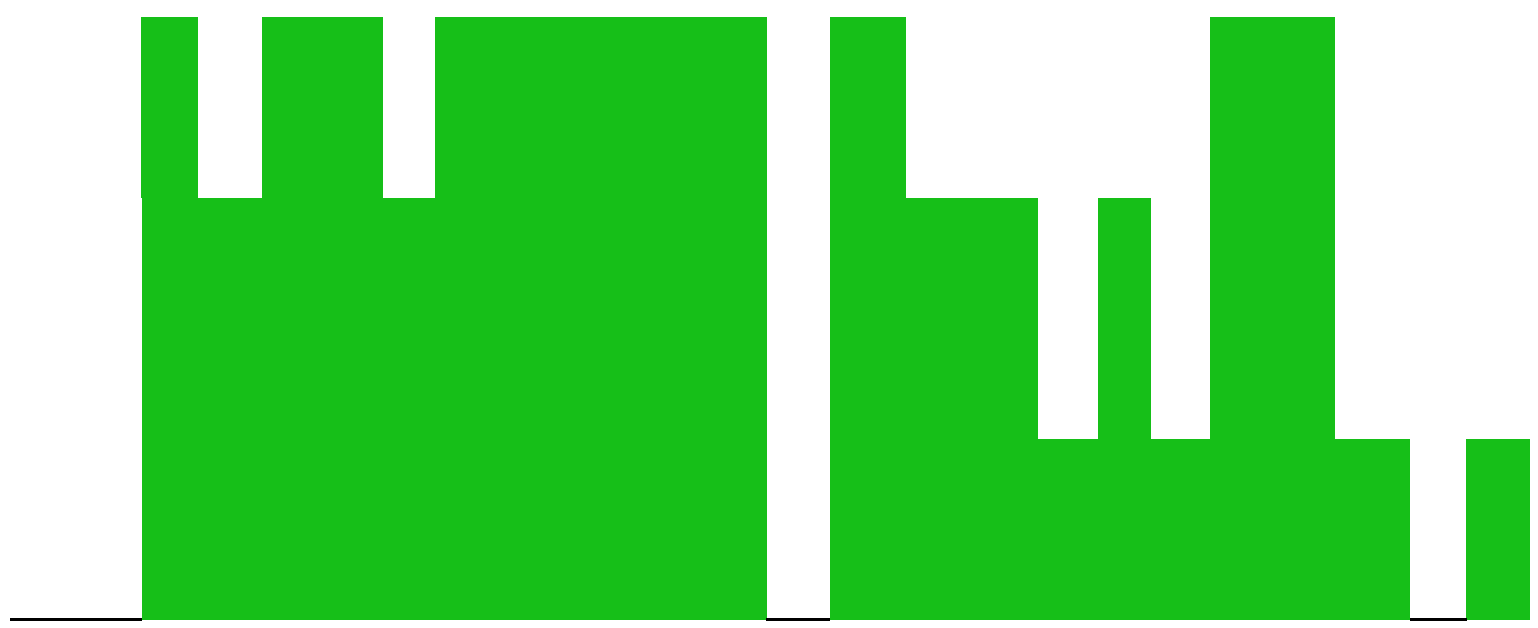

GLI2 aa418-aa437
R426Q

Homo sapiens	W	A	G	P	T	P	H	T	R	N	T	K	L	P	P	L	P	G	S	G
Mus musculus	W	A	G	P	A	P	H	T	R	N	T	K	L	P	P	L	P	V	N	G
Xenopus laevis	F	I	N	P	P	P	P	A	R	N	T	R	L	P	T	I	T	A	N	G
Danio rerio	W	A	N	A	P	P	Q	V	R	N	T	K	L	P	P	I	P	S	I	D
									*											

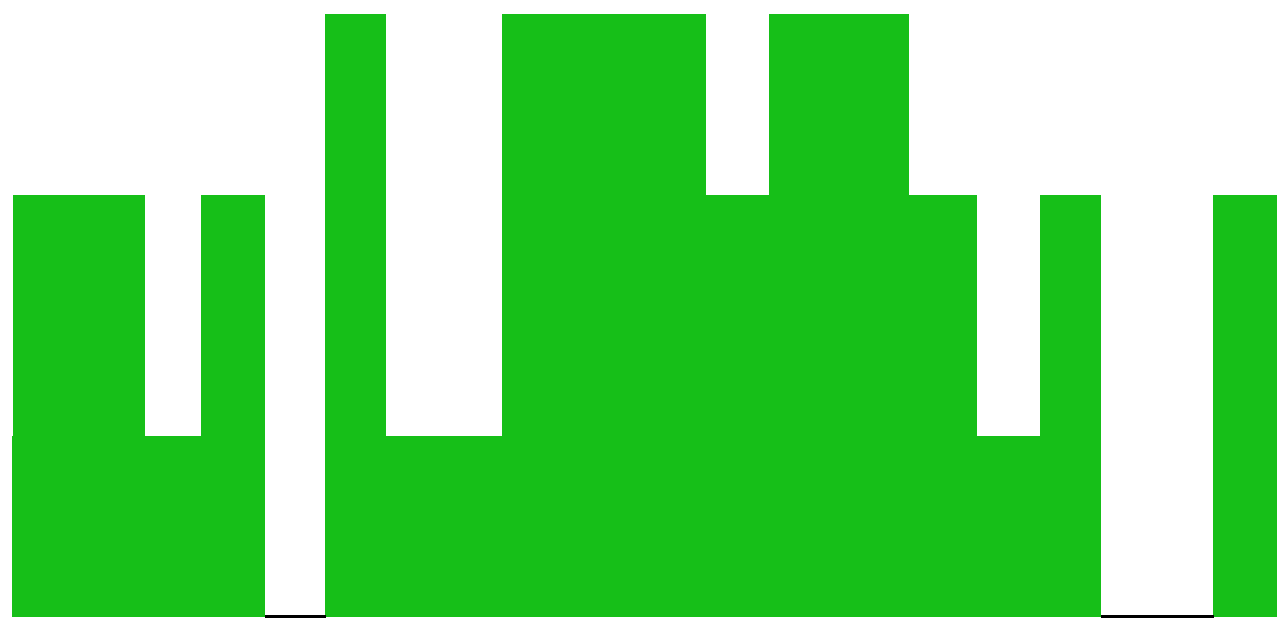

GLI2 aa771-aa788
G778D

Homo sapiens	L	P	S	P	G	L	H	G	Q	R	R	M	V	A	A	D	S	N
Mus musculus	L	P	S	P	G	L	Q	G	H	R	R	L	A	A	A	D	S	N
Xenopus laevis	P	F	H	H	N	T	P	H	R	A	P	G	A	Y	S	S	R	N
Danio rerio	S	Q	Q	Q	Q	F	Y	G	Q	R	R	M	G	M	A	G	I	N
								*										

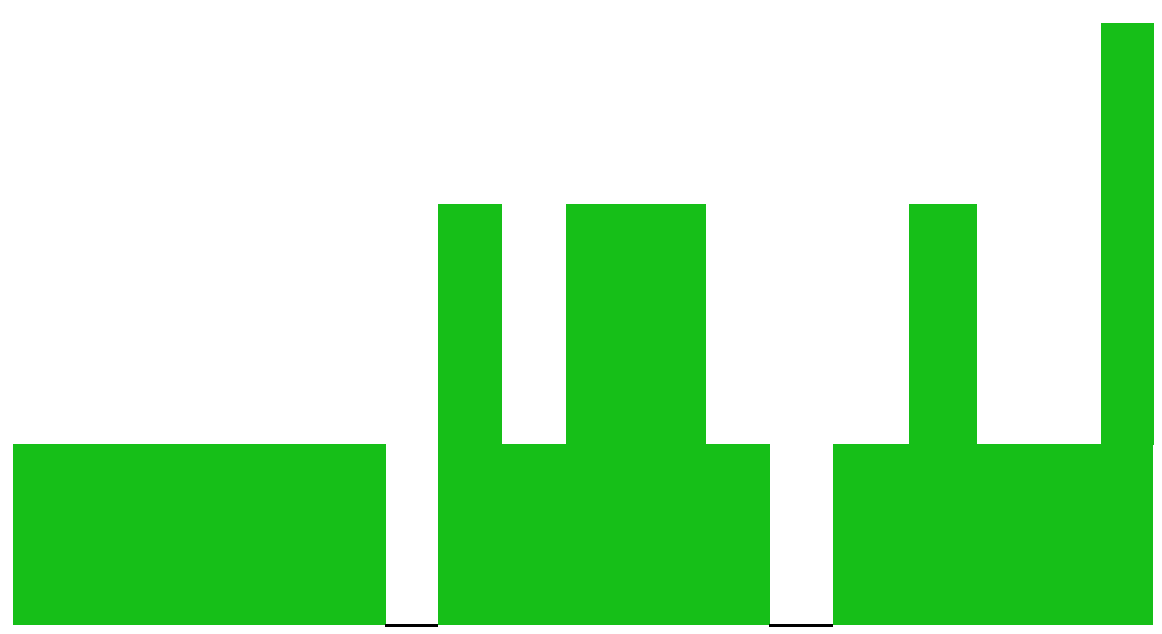

GLI2 aa1201-aa1223
S1213Y

Homo sapiens	L S P S L L H S L S Q N S S R L T T P R N S L
Mus musculus	L S P T L L H N L S Q N S S R L T T P R N S L
Xenopus laevis	L S P G L L H S F S Q T S S R L T T P R N S L
Danio rerio	L S P G L L Q S I S Q N S S R L T T P R N S V
	* (at position 13)

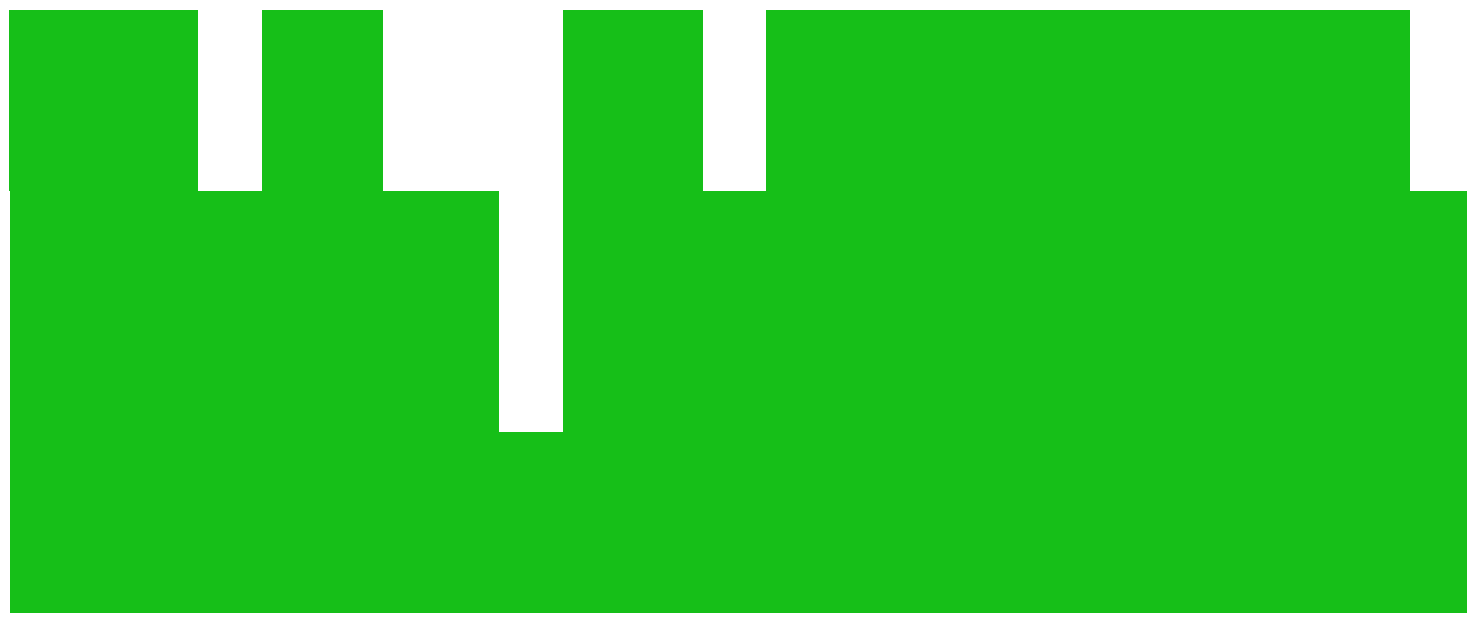

LHX8 aa210-aa240
E221A

Homo sapiens	Y D C M L H N L K R E V E N G N G I S V E G A L L T E Q D V
Mus musculus	F D C M L D N L K R E V E N G N G I S V E G A L L T E Q D V
Anopheles gambiae	Y - - - - - - - M D T V E D G S N S S D D G C - - - S S D G
Caenorhabditis elegans	F E L E - - - - - - - V E D G S G V S S Q K A - - - - - - -
Drosophila melanogaster	Y - - - - - - - L E T V E G G T T S S D E G C - - - D G D G
	* (at position 11)

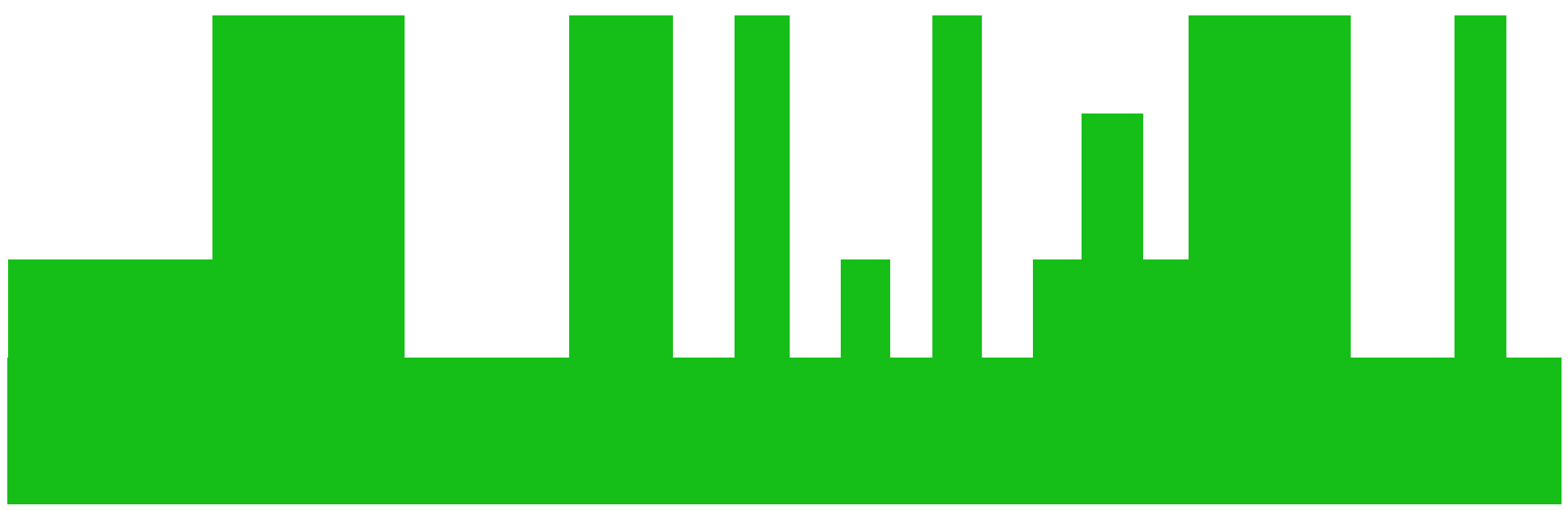

JAG2 aa578-aa610

M597I

Homo sapiens	C P G G A C R V I D G C G S D A G P G M P G T A A S G V C G P H G
Mus musculus	C P G G A C R V I D G C G F E A G S R A R G V A P S G I C G P H G
Rattus norvegicus	C P G G A C R V I D G C G F E A G S R A H G A A P S G V C G P H C
Gallus gallus	C K N N S C K V I D S C T I E V F S N T T Q E G I R F I S S N V C
Danio rerio	C K M T P C Q V I D S C T I A V A S N S S D G G V R H I N S N V C
Tetraodon nigroviridis	C R T T T C K V I D S C T V A V A S N S T P G G E R Y I S S N V C
Xenopus laevis	C R T T P C E V I D S C T V A V A S N S T P E G V R Y I S S N V C
Tribolium cantaneum	N P P C D D E V E S C I A V G V G S G R N S A P S I C G E H G H C
Apis mellifera	N P P C D D E V E S C I A V G V G S G R N S A P S I C G E H G H C
Anopheles gambiae	C L S S P C R N G A V C V E A D G Y G L T G R Y A
	*

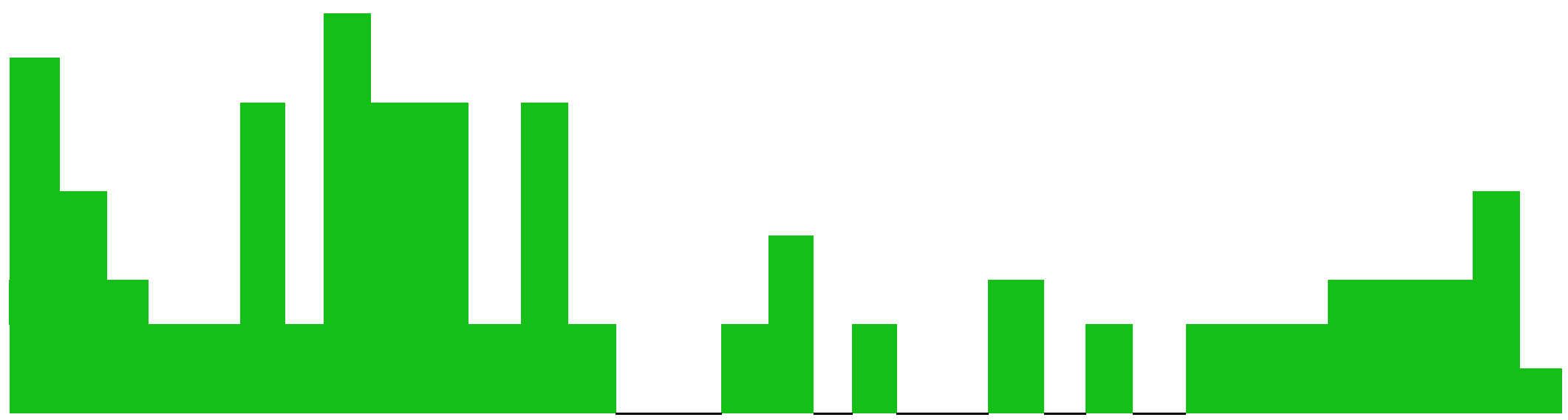

JAG2 aa636-aa672

A657H

Homo sapiens	N I D D C L G Q P C R N G G T C I D E V D A F R C F C P S G W E G E L C D
Mus musculus	N I D D C M G Q P C R N G G T C I D E V D S F R C F C P S G W E G E L C D
Rattus norvegicus	N I D D C M G Q P C R N G G T C I D E V D S F R C F C P S G W E G E L C D
Gallus gallus	N I N D C L G K P C K N G G T C I D E V D S F K C F C S S G W E G E L C D
Danio rerio	N V N D C V S N P C R N G G T C I D G I S S F Q C F C P D G W E G D L C S
Tetraodon nigroviridis	N I N D C E S N P C H N G G T C I D K V S V Y Q C I C A D G W E G D H C Q
Xenopus laevis	N I N D C E S N P C N N G G T C I D G V N S Y K C I C S N G W E G I Y C E
Tribolium cantaneum	N I N D C K V N P C E N G G T C V D K I N A F Q C I C K E G W E G A L C N
Apis mellifera	T I D D C A S D P C Q N G G T C I D Q L E G F V C K C R P G F V G L Q C E
Anopheles gambiae	N W D E C W S D P C L N G G T C I D G V A S Y N C T C P D G F L G L N C E
Caenorhabditis elegans	N W D E C W S D P C I N G G T C I D G V A S Y N C T C P D G F L G Y E G K
Drosophila melanogaster	N I D D C L G E I K C Q N N A T C I D G V E S Y K C E C Q P G F S G E F C
	* (position 22)

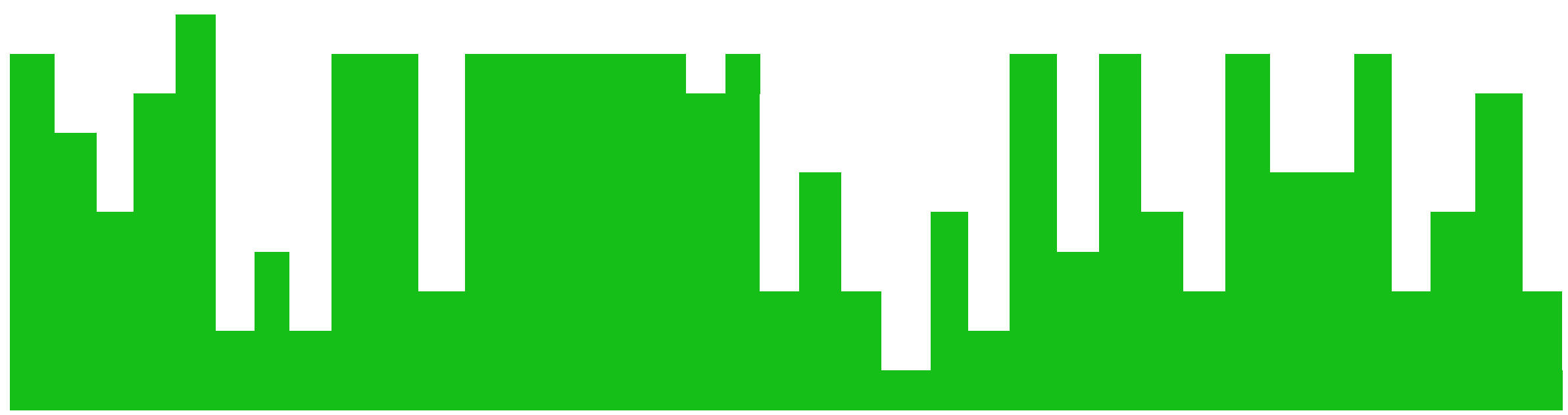

MSX2 aa53-aa74
S63C

Homo sapiens	M	S	D	-	K	K	P	P	K	E	A	S	P	L	P	A	E	S	A	S	A	G	A
Mus musculus	M	S	D	-	K	K	P	P	K	E	S	P	A	V	P	P	D	C	A	S	A	G	A
Canis familiaris	M	S	D	-	K	K	P	P	K	G	A	S	P	R	P	A	D	S	A	S	A	G	A
Gallus gallus	M	S	D	-	K	K	P	P	K	E	L	P	L	A	A	A	G	G	N	A	D	G	A
Japanese quail	M	S	D	-	K	K	P	P	K	E	L	P	L	A	A	A	G	S	S	A	D	G	A
Xenopus tropicalis	M	A	D	K	R	V	P	K	E	A	P	H	L	R	G	V	D	A	S	A	A	G	S
												*											

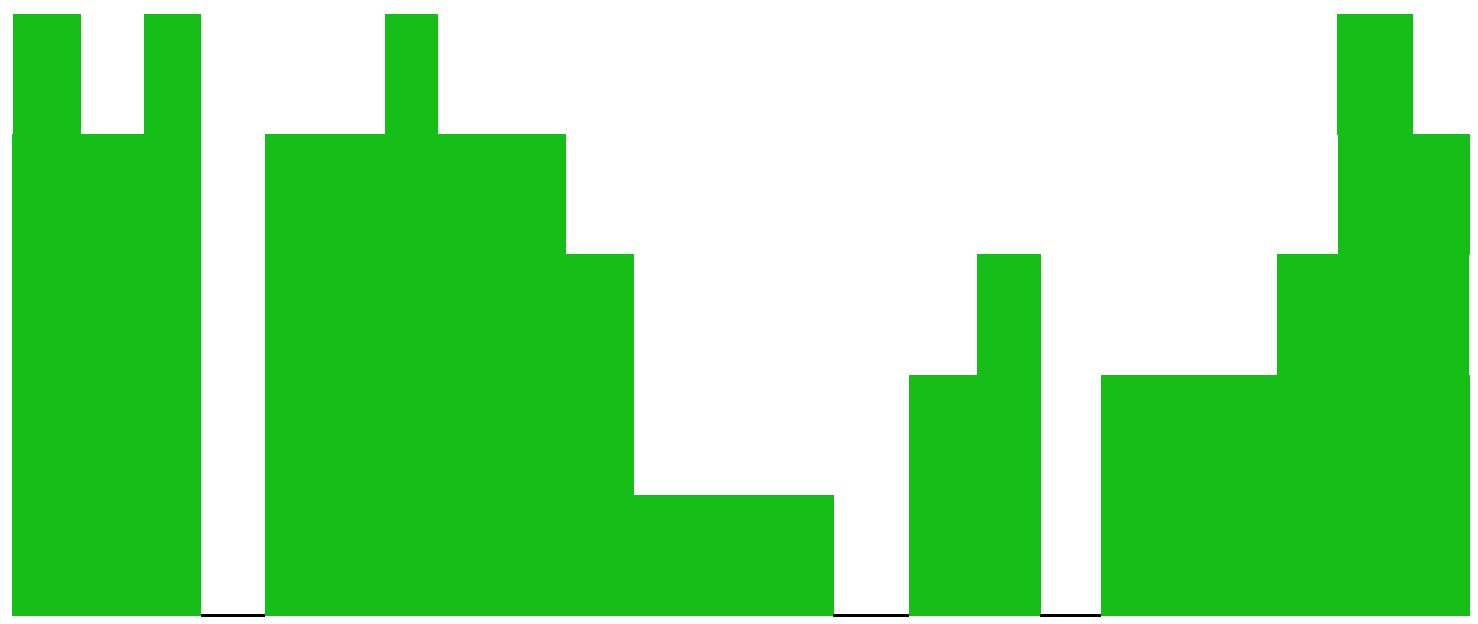

SATB2 aa181-aa198
T190A

Homo sapiens	E	L	L	K	E	M	N	Q	S	T	L	A	K	E	C	P	L	S
Mus musculus	E	L	L	K	E	M	N	Q	S	T	L	A	K	E	C	P	L	S
Rattus norvegicus	D	L	L	K	D	M	Q	S	S	S	L	A	K	E	C	P	L	S
Equus caballus	D	L	L	K	D	M	N	Q	S	S	L	A	K	E	C	P	L	S
										*								

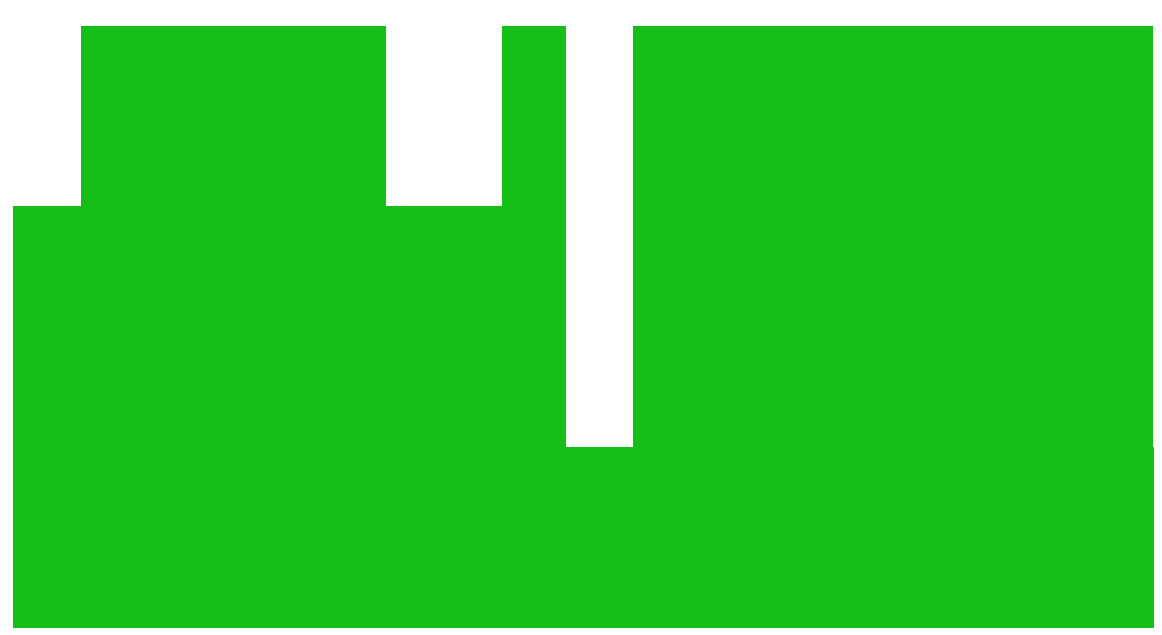

SKI aa361-aa393
A388V

Homo sapiens	G S S N K S L G C V H P R Q R L S A F R P W S P A V S A S E K E L
Mus musculus	G S S N K S L G C T H P R Q R L S A F R P W S P A V S A S E K E T
Gallus gallus	S S S N K S I G C V H P R Q R L S A F R P W S P A V S A N E K E L
Danio rerio	A S A N K G L N C I Q P R Q R P S A F R P W S P N I S V S D K D G
Xenopus laevis	- N T N K N T G Y V H P R Q R L S A F R P W S P A I S A N D K E L
Oreochromis aureus	- T P N K G L N C I Q S R Q K P S A F R P W S P H I S A G D K E P
Crocodylus niloticus	N S S N K S I G C V H P R Q R L S

*

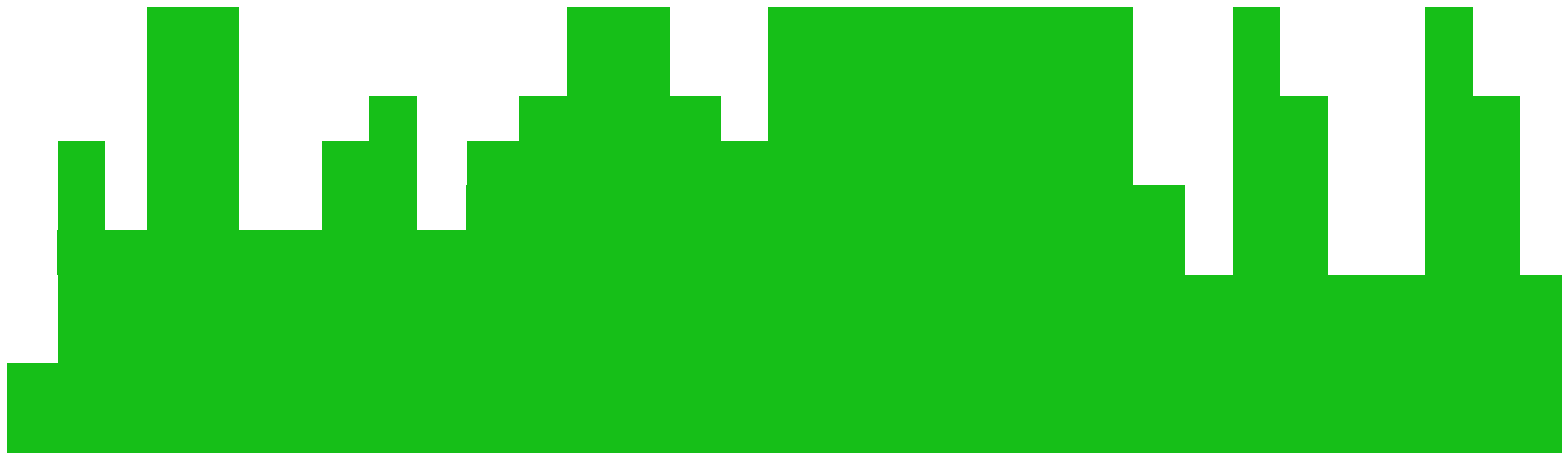

SPRY2 aa1-aa30
D20A

Home sapiens	M E A R A Q S G - N G S Q P L L Q T P R D G G R - Q R G E P D P
Mus musculus	M E A R A Q S G - N G S Q P L L Q T A H D S G R - Q R G E P D P
Rattus norvegicus	M E A R A Q S G - N G S Q P L L Q S P H D S G R - Q R G E P D P
Gallus gallus	M E T R V Q H G - S G S Q A L L Q A R R D S G R - P H G E P D L
Xenopus laevis	M E T R V Q N G - N V S Q P L L P A R R D N G R - L H S D T D P
Danio rerio	M E T R T Q N G G S G S S G L L R A L R D S G R P Q T G D S E P

*

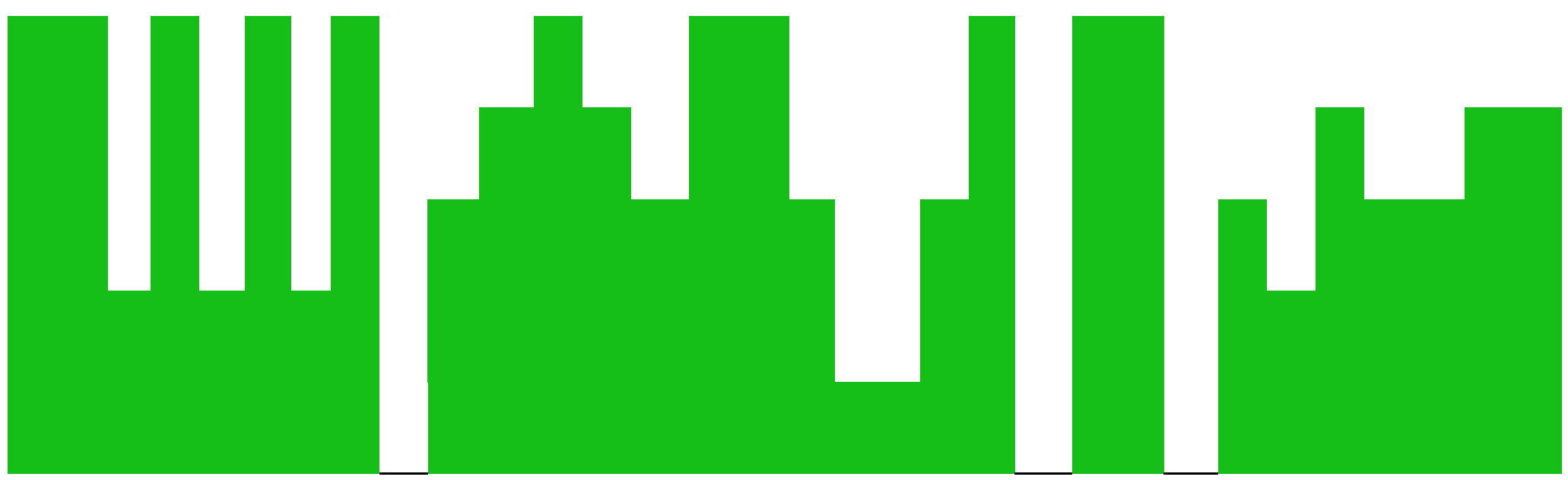

SPRY2 aa61-aa92
K68N

Home sapiens	V V P R P G L K P A P R P S T Q H K H E R L H G L P E H R Q P P
Mus musculus	V V P R P G L K P A P R P S T Q H K H E R L H G L P E H R Q P P
Rattus norvegicus	V V P R P G L K P A P R P S T Q H K H E R L H G L P E H R Q P P
Gallus gallus	V A P R P G V K S A P R S A S Q P K S E R P H G L P E H R H F G
Xenopus laevis	V A P R P V I K S A P R Q T T Q Q K N E R I H V V N E Q R P F V
Danio rerio	V A P R P - - - P A S E Q K T D S Q A S S P T S T S S I E L P E
	* (under K)

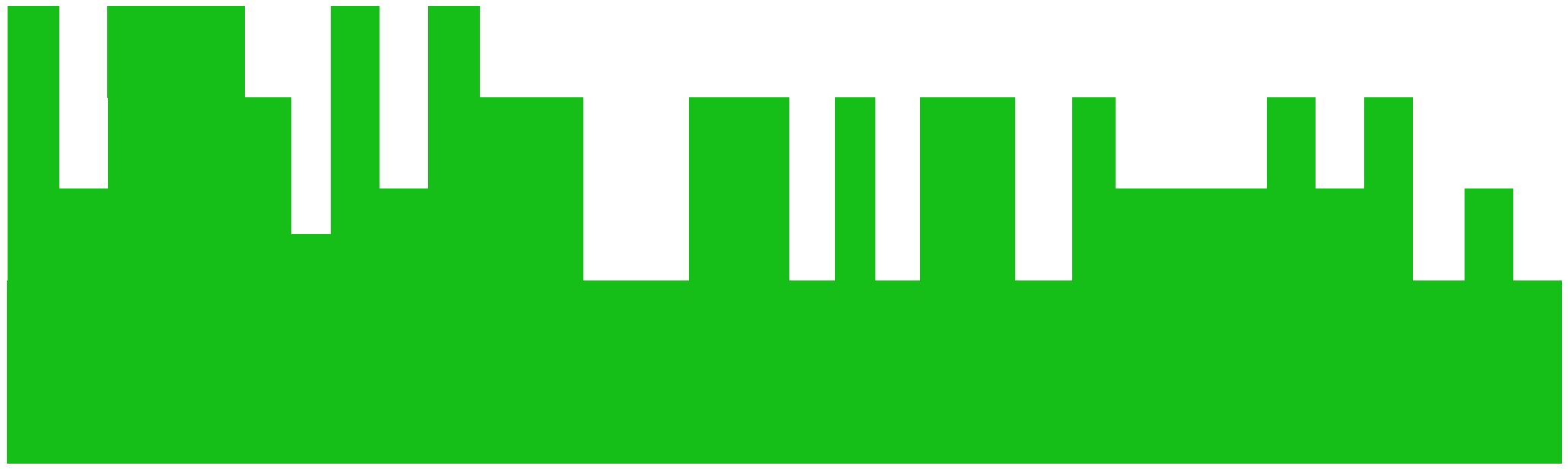

TBX10 aa346-aa362
R354Q

Homo sapiens	A P Y P L P N I R A D R D Q G G L
Mus musculus	A P Y P L P N I S T A G D Q E D P
	* (under R/S)
