# Supplementary material for: Medical Sequencing of Candidate Genes for Nonsyndromic Cleft Lip and Palate
Source: PLoS Genet. 2005 Dec 2;1(6):e64. doi: 10.1371/journal.pgen.0010064 (PMC1298935; doi:10.1371/journal.pgen.0010064)
Supplement: Supplementary file 2 [file pgen.0010064.st001.pdf]

Primers and PCR conditions.

GENE	REGION	PRIMER SEQUENCE	ANNEALING TEMP	PCR CONDITIONS
ISGF3G	exon 1	5' - AGA TGC TGC TGC CCT CTA GT - 3'	53°	94°C - 3 min.
(IRF9)		5' - CCT CCT GAC CTT TGC CTA CA - 3'		94°C - 30 sec. } 35 cycles
	exon 2	5' - TGA TGG CAG AAA GGA AAC AA - 3'	53°	__°C - 30 sec. } 35 cycles
		5' - TGC AAA TGA TGC AGC TAG TTT T - 3'		72°C - 30 sec. } 35 cycles
	exon 3	5' - CAG AGC ACA GAC CCT GCA TA - 3'	53°	72°C - 7 min.
		5' - AGC TAT AAG CCT GGG GGA AG - 3'		4°C - hold
	exon 4	5' - GGA TAG ATG TGC AGG CTT CC - 3'	53°	
		5' - GAG ACG GGT TCT CAC CAT GT - 3'		
	exon 5-6	5' - CCT GGG CAA CAA GAG TGA A - 3'	53°	
		5' - TCT CGC CAC CTA GAA ACC AC - 3'		
	exon 7	5' - GAG GGG AGG TGG AGT TGT TC - 3'	59°	
		5' - AGG TAG CAG GAC CCA ACA AG - 3'		
	exon 8	5' - CCA AGT ACT TCT GAC CTT GCA G - 3'	53°	
		5' - AGC ATC TCA GTG GGT GCA T - 3'		
	exon 9	5' - CAA AGG GGG TCT CCC AGT - 3'	53°	
		5' - AGC AGG AAG AGA ACC CCA GT - 3'		
JAG2	exon 2a	5' - TCC CGC TGC CTT ATT TTT AG - 3'	57°	94°C - 3 min.
		5' - ACC TTG GCC TGG TAC TCC TT - 3'		94°C - 30 sec. } 35 cycles
	exon 3	5' - CTT GAG CTG GGT CAT TTT T - 3'	56°	__°C - 45 sec. } 35 cycles
		5' - CAC ATG ATC CTC AGG GTA AA - 3'		72°C - 3 min. } 35 cycles
	exon 4-5	5' - GTT TCT CTG GCC TGT GCT - 3'	58°	72°C - 3 min.
		5' - AGT TGG GAG GGC AAA GAC - 3'		4°C - hold
	exon 6-7	5' - CAC AGC TCA CTG GGT CTT - 3'	58°	
		5' - CTC CCC AGG GAA CCA GTC - 3'		
	exon 8	5' - GGA GGA GTG CTT GTG GAA AG - 3'	58°	
		5' - CCC TAC TCA CGT GCA GAC AC - 3'		
	exon 9	5' - CCA CCT TTC TCC ACC CTC T - 3'	63°	
		5' - GTT CCC ATG CAC TCG CTC - 3'		
	exon 10-12	5' - ACG GCT CCC ACC TTG TCT - 3'	60°	
		5' - CCT ACA GCT CCC AGA GCA - 3'		
	exon 13-15	5' - GGC CTA GGC TCT CAC AGG - 3'	59°	
		5' - GTC CAT CTC AGG GTG ATA AG - 3'		
	exon 16-17	5' - CAG CTC CAG TGC TTC CTC - 3'	61°	
		5' - CAG ACA GGG CAG ACA GAC - 3'		
	exon 18	5' - CCC TCC TCC TCC TCT CCT TA - 3'	63°	
		5' - CAG GGA TGT CAT CTG GGT G - 3'		
	exon 19-20	5' - TAC TGA TCC CCT GCG TTC - 3'	58°	
		5' - CAG AAG AGA GGC AGG AAG TA - 3'		
	exon 21-22	5' - CTG CTC CTC ATT GTC CTG - 3'	60°	
		5' - CTC CTG ATC TCA CAG AGT GG - 3'		
	exon 23	5' - GTG GGT TCT CAT CTG TGT G - 3'	59°	

		5' - CTC TGC AAG CTC AGA TGC - 3'		
	exon 24	5' - CCA GGG ATG TAG GTG TCA GG - 3'	61°	
		5' - CCA CTG GGC ACT CAC CAC - 3'		
	exon 25	5' - GTT CTT TGC CGC AGT CCT T - 3'	58°	
		5' - CTG AGT TGC CTG CCT GGT - 3'		
	3'UTR-1	5' - CTG GGT GTG TGG CTA GTG - 3'	55°	
		5' - CCA GGA TCT TTG GTG AAT TT - 3'		
	3'UTR-2	5' - ATG AGG AGG ACG AGG ATC T - 3'	61°	
		5' - CAC AGC CAT GGG TCT CAC - 3'		
	3'UTR-3	5' - AAA ACC ACC AAA AAC AAA AA - 3'	55°	
		5' - ACA GCC ACA GGT CCC ATT - 3'		
LHX8	exon 1	5' - CCC CTT CCT TCA ACA ACA AA - 3'	55°	94°C - 3 min.
		5' - TCT TGA GGT CAC GAT TCT CG - 3'		94°C - 45 sec. }
	exon 2	5' - CGA GAA TCG TGA CCT CAA GA - 3'	56°	__°C - 45 sec. } 35 cycles
		5' - TAG ACC CCT CCT CCC CTT C - 3'		72°C - 1 min. }
	exon 3	5' - GAA GGG GAG GAG GGG TCT A - 3'	55°	72°C - 2 min.
		5' - AAC TGT TGC ACA CAC ACT TGC - 3'		4°C - hold
	exon 4	5' - CTG AGG GCC TTC ATT AGC TC - 3'	55°	
		5' - GCA CTC ACC AGT CCC TCT TC - 3'		
	exon 5	5' - TGG GGT AAG ATA ATG TGC TGG T - 3'	52°	
		5' - TTC CTG TTT CAC TGT GTC AAA A - 3'		
	exon 6	5' - TAC CTG TAA CGG GCC TCA TC - 3'	55°	
		5' - CCC CAG CAA ATA GAC CAA AA - 3'		
	exon 8	5' - TTA TGG CAA ATG CAG ACA GC - 3'	56°	
		5' - TAT AAA GGA AGG GGG CGT GT - 3'		
	exon 9	5' - ACA CGC CCC CTT CCT TTA TA - 3'	57°	
		5' - ATA CAT GGC ACT GCT AGG GC - 3'		
NAT1	exon 1	5' - TGGGTTTGGACGCTCATA - 3'	56°	94°C - 7 min.
		5' - CAGAGTCTTGAACTCTATT - 3'		94°C - 1 min. }
				__°C - 1 min. } 35 cycles
				72°C - 1 min.30sec. }
				72°C - 10 min.
				4°C - hold
P63	5'UTR	5' - TGT AAA ACG ACG GCC AGT CTG GTA AGA ATC GAG	57°	94°C - 5 min. (Ex 1,3,4,5,7,12)
		5' - CAG GAA ACA GCT ATG ACC TGG GAA GAT TCG CTT		94°C - 30 sec. }
	exon 1	5' - TGT AAA ACG ACG GCC AAG AAA CGC TCC GCC TCT -	57°	__°C - 45 sec. } 30 cycles
		5' - CAG GAA ACA GCT ATG ACC AAA TGG GAA GAT TCG CTT CA - 3'		72°C - 1 min.30sec. }
	exon 3	5' - TGT AAA ACG ACG GCC AGT TCC ATT GGA GTG GAG GAG TC - 3'	57°	4°C - hold
		5' - CAG GAA ACA GCT ATG ACC CAG CCA CCA CAG AAA ACA TT - 3'		

	exon 4	5' - TGT AAA ACG ACG GCC AGT GTG AAG TGC TTC CGA CGT G - 3'<br>5' - CAG GAA ACA GCT ATG ACC TTT CCA CCA TGA ACA TGG AAT - 3'	57°	
	exon 5	5' - TGT AAA ACG ACG GCC AGT AGC CTC AGC TTC TTC ATG CT - 3'<br>5' - CAG GAA ACA GCT ATG ACC ATG CCC ACA GAA TCT TGA CC - 3'	57°	
	exon 7	5' - TGT AAA ACG ACG GCC AGT TTG CCC TTT TAG GAG GAA GC - 3'<br>5' - CAG GAA ACA GCT ATG ACC GTG AAA TGC CTT GAG GAC CA - 3'	57°	
	exon 9	5' - TGT AAA ACG ACG GCC AGT CAG CAA GAT AAC GGG CTT TC - 3'<br>5' - CAG GAA ACA GCT ATG ACC TCC TTT CCC ATT GTC ACA GA - 3'	57°	
	exon 10	5' - TGT AAA ACG ACG GCC AGT TGT TTC CTT CTT TCC TTC TGC - 3'<br>5' - CAG GAA ACA GCT ATG ACC TGT GTA TGG ACC AGG CAT ATA GA - 3'	57°	95°C - 5 min. (Ex.9,10,11,13,14,15)<br>95°C - 15 sec.
	exon 11	5' - TGT AAA ACG ACG GCC AGT AGC CTC GCA GTC AGT TTC TC - 3'<br>5' - CAG GAA ACA GCT ATG ACC GTC AGA AAC CCA AGC CAC TC - 3'	57°	__°C - 30 sec. 35 cycles<br>68°C - 1min.
	exon 12	5' - TGT AAA ACG ACG GCC AGT AAG CAG AGA AAG GGG AGG AA - 3'<br>5' - CAG GAA ACA GCT ATG ACC AAC TCC AAC CAC AGG ACT GC - 3'	57°	68°C - 3 min.<br>4°C - hold
	exon 13	5' - TGT AAA ACG ACG GCC AGT AAT CGC TGA TCT GCA GGA AT - 3'<br>5' - CAG GAA ACA GCT ATG ACC ATA GGT TGA GGC CTG GGA AT - 3	57°	
	exon 14	5' - TGT AAA ACG ACG GCC AGT TGC CTT TGT GCT TAG TTC C - 3'<br>5' - CAG GAA ACA GCT ATG ACC AAG CAG GAG TGC TTT TAG GG - 3'	57°	
	exon 15	5' - TGT AAA ACG ACG GCC AGT GCC TTA TCC CAT TTC TTC CTC - 3'<br>5' - CAG GAA ACA GCT ATG ACC TAG TGG CTT TGT GCC TTT GA - 3'	57°	
PAX9	exon 2	5' - CAG TCC CCG GAT GCG TAG - 3'<br>5' - TCC CTG AGG CTG CAG ATA CT - 3'	61°	94°C - 30 sec.<br>__°C - 1 min. 35 cycles
	exon 3	5' - GCA GCC TTA AGG GTT CCT TC - 3'<br>5' - ACG AAG GAT CTG GCT CGT AG - 3'	62°	72°C - 1 min.<br>4°C - hold

	exon 4	5' - TTG TTT AAG TGG CAA GAC TGT GA - 3'<br>5' - TCC TAA AGT CAT GTC AGT TTT TCT G - 3'	63°	
SATB2	exon 2	5' - CTA TCA CGT GTA TGA GCG G - 3'<br>5' - TAA TCA ACC TGA ACC CTG AC - 3'	56°	94°C - 5 min.<br>94°C - 45 sec. } 35 cycles<br>__°C - 45 sec. }<br>72°C - 45 sec. }<br>72°C - 7 min.<br>4°C - hold
	exon 3	5' - CTG TTG CTT CCC TTC TCA TC - 3'<br>5' - TAT TTC AGA AAA CAT ATT CTT CC - 3'	53°	
	exon 4	5' - GAG TGG GCC TTG GAT GTT G - 3'<br>5' - TGT CCA GAA ATT AAT AGA CAG G - 3'	58°	
	exon 5	5' - ATT CTT TAG CAT TTC TTC TGA AG - 3'<br>5' - TGA TAG AGA GTC TAC CAA TGG - 3'	55°	
	exon 6	5' - AGT TCA GTT TAC AGG AGA GTG - 3'<br>5' - CGT AAA TTG TAT CTA AAA TGA GC - 3'	55°	
	exon 7	5' - AAA GTA TGA TAA TTC AGT TCT AC - 3'<br>5' - TAA CCT ATC TCA ATT CTG TCC - 3'	52°	
	exon 8	5' - GGA AGA CAG AAA CCA CAC AC - 3'<br>5' - GGG AAA ACA CAG TAA CTA GTG - 3'	55°	
	exon 9	5' - GAT AAT TTC TGC ACA GAT GGC - 3'<br>5' - GTT TCT TGG GGG TTA TTA CTG - 3'	55°	
	exon 10	5' - ATG TAC TGT GAT GGC ACT GG - 3'<br>5' - GTG GTG TGT GCC ACT TGG - 3'	58°	
	exon 11	5' - CTT ACC TCC AGT GCT AGA TAG - 3'<br>5' - TAA AGA AAT GAA AGC AGA AAA TCC - 3'	57°	
SKI1	exon 1a	5' - CTG GAG CAG TTC CAC CTG A - 3'<br>5' - TGC CGA AGC ACT CGT GGT AC - 3'	59°	94°C - 5 min. (Ex.1a,2-3,4-5)<br>94°C - 30 sec. } 35 cycles<br>__°C - 30 sec. }<br>72°C - 2 min. }<br>72°C - 3 min.<br>4°C - hold
	exon 1b	5' - TCC TCA AAG TCA TGG GCA TC - 3'<br>5' - GGC CTT CCT ACC AAA CTC TCT G - 3'	55°	
	exon 2-3	5' - TGA CTT GTC CCT CAG CAC AG - 3'<br>5' - AGG AAC CAA CAC CGA GAC TG - 3'	57°	
	exon 4-5	5' - GTG GCG CAG GAT GTG TCT - 3'<br>5' - GTG CAG GCT GTG CTC ACT - 3'	57°	
	exon 6	5' - CTC CAG GGC ACA TTG TCA G - 3'<br>5' - GAT CTG AAA GGG AGC CGA GT - 3'	55°	94°C - 3 min. (Ex.1b,6,6-7,7)<br>94°C - 45 sec. } 35 cycles<br>__°C - 45 sec. }<br>72°C - 45 sec. }<br>72°C - 3 min.<br>4°C - hold
	intron 6 - exon 7	5' - GTC TCG TGA GCC TGT GTC CT - 3'<br>5' - AAT GTT CCA GCA AGC AGA GG - 3'	55°	
	exon 7	5' - TGT AGT TTT GGA ACC CAC TGC - 3'<br>5' - AGA ATC ACG GGG TGC AGA - 3'	55°	
SPROUTY1	exon 1	5' - AGTGGAGGTGGAGGTGGA - 3'<br>5' - CAGTTCCTTGCAAAAGTCAACA - 3'	55°	94°C - 5 min.<br>94°C - 45 sec. } 35 cycles<br>__°C - 45 sec. }<br>72°C - 45 sec. }<br>72°C - 7 min.<br>4°C - hold
	exon 2a	5' - GGCAATTTGTGATTTGACAGG - 3'<br>5' - CAGTGCTGGTTGATCTGCTC - 3'	50°	
	exon 2b	5' - ACGAGCACAGACACACAAGC - 3'<br>5' - ATAGGAATCCCCTTCGTCGT - 3'	53°	
	exon 2c	5' - TACCATCCTGTTTGGCCTGT - 3'	56°	

		5' - TCAGGAGGTACAACCCACCT - 3'		
	exon 2d	5' - TGTCTATTGTAAGCTGGAGAGCTG - 3'	55°	
		5' - CTGGATGTGGGTGTATGTTCA - 3'		
	exon 2e	5' - TGGGCTTTTGTGTAGCCTTT - 3'	54°	
		5' - GAGAAGCATGGGGTGAAGAA - 3'		
SPROUTY2	exon 1	5’ - GCA CCA CTT GGC GAT CTG - 3'	57°	94°C - 5 min.
		5’ - GGC TGC ACC TAC TCC ATG TT - 3'		94°C - 30 sec. } 35 cycles
	exon 2a	5’ - GGG TGG AGA GTG GGA AGT ATC - 3'	57°	__°C - 30 sec. } 35 cycles
		5’ - GAG TGC TGG AGC CTA GGA G - 3'		72°C - 30 sec. } 35 cycles
	exon 2b	5’ - GCC TAC TGT CGT CCC AAG AC - 3'	57°	72°C - 7 min.
		5’ - TCC CAT AGT CAA TCA CGT TCT G - 3'		4°C - hold
	exon 2c	5’ - AAG GAG TGC ACC TAC CCA AG - 3'	54°	
		5’ - TGA TGC TAT GTT GGT TTT TCA A - 3'		
TBX1	exon 1	5' - GAA ATG GGC GTC TTG TCT TC - 3'	58°	94°C - 5 min.
		5' - CAG CAG GAT AGG GCT CCT C - 3'		94°C - 30 sec. } 35 cycles
	exon 2	5' - CCA TGA CGC CAT AAT CCT CT - 3'	55°	__°C - 30 sec. } 35 cycles
		5' - CTT CCT CTC TGC CCC AGA AT - 3'		72°C - 30 sec. } 35 cycles
	exon 4	5' - GCC AAG CTC CCA GTT GAG TA - 3'	57°	72°C - 7 min.
		5' - CTG AGA GGG CAT GGA GTA GG - 3'		4°C - hold
	exon 5	5' - GCA GCA GAG GGT TCA ATC TC - 3'	59°	
		5' - TAG CCT CGC AGG GAC TCT AA - 3'		
	exon 8	5' - CCC TGA GGA CTG GTG AGT GT - 3'	58°	
		5' - GCG GAG AGA AGG CTC TTG - 3'		
	exon 9	5' - GAT GGT GTG TGA GGC TGA TG - 3'	59°	
		5' - AGC AGC ATA ACA GCC ATT CT - 3'		
	exon 10	5' - GCT GCT CTG TTT GAG GTG GT - 3'	56°	
TBX10	exon 1	5' - ACC TCA GGA CGT GGA CGA - 3'	55°	94°C - 3 min.
		5' - GTC CAG CCC CAA CTT TGA T - 3'		94°C - 45 sec. } 35 cycles
	exon 2	5' - ACT ACC GTC AAG GAG CTT GC - 3'	53°	__°C - 30 sec. } 35 cycles
		5' - CAG GAT CTT CAC CTG GAA GG - 3'		72°C - 45 sec. } 35 cycles
	exon 3	5' - TGA GAT GAT CGT CAC CAA GG - 3'	54°	72°C - 3 min.
		5' - GGC TGG TGG ATG ACA GGA TA - 3'		4°C - hold
	exon 4	5' - TTG GGT TCC TTA GCA AGG TG - 3'	55°	
		5' - CTT TTC CAC CTG CCT GGA C - 3'		
	exon 5	5' - CTC CAG CTC AGA CCC TGT G - 3'	58°	
		5' - GGG CAG TAG TCT GTG CCA GT - 3'		
	exon 6-7	5' - CCT CCT GAC TGC CGG TTC - 3'	55°	
		5' - CCT TCC GCC CAC TGT GTA - 3'		
	exon 8a	5' - ATT CCA GGC AGA GGG AAA AG - 3'	55°	
		5' - CGG TGT AAG GTC CAG GGT AG - 3'		
	exon 8b	5' - ACT CCC AGT GAT GCC AGA AG - 3'	54°	
		5' - GGG CTG GAG TCA CCA CTT T - 3'		
TGFA	5' UTR	5' - CCG CCT AGA GCC TGG AAG - 3'	57°	95°C - 5 min.

		5' - AGC CGA GGG GAC CAT TTT AC - 3'		95°C - 15 sec. } 35 cycles
	exon 1	5' - GGT AGC CGC CTT CCT ATT TC - 3'	57°	__°C - 30 sec.
		5' - GCA GCC AGG TAA GCA GGT AG - 3'		68°C - 1 min.
	exon 2	5' - TAC CGG CCA CTC TGT GAA AC - 3'	57°	68°C - 5 min.
		5' - CTT GGG CCT GCA GTT AAG AC - 3'		4°C - hold
	exon 3	5' - TGA GGG GAA ATT TGT TTT GC - 3'	57°	
		5' - ATG CTA GAG GGC AGA GAC CA - 3'		
	exon 4	5' - CAG GAT TTG GCA CAG TCA CA - 3'	57°	
		5' - ATA CTG GGG ATG TCC CTG GT - 3'		
	exon 5	5' - CAG GTC AGC TTG CAG AAA CA - 3'	57°	
		5' - TTC TCC TCT CTT CCT GCT TCA - 3'		
	exon 6	5' - GGT CCC TGA GAC TGA ACC AG - 3'	57°	
		5' - ATG GCT GGC AGA AGA CAA CT - 3'		
	3' UTR-J	5' - GGT CTG AAG AGC CCA GAG GAG G - 3'	63°	
		5' - GCA CAC ATG TGG ACT CAG ACA CC - 3'		
	3' UTR-K	5' - GAG ACG GAC TCC TGT TCA CCT AGG - 3'	60°	
		5' - CAA GGG TGG CGA TAG CTT GGG - 3'		
	3' UTR-M	5' - GAG GCT GGG TTA CTG GTA GG - 3'	60°	
		5' - GAC TGG TCC CCC TTT CAT GG - 3'		
	3' UTR-N	5' - GGG TTC ACT TCC TCG TGA GC - 3'	53°	
		5' - GGT TGA CCA CAT AGT GAG GTG AC - 3'		
	3' UTR-O	5' - CAT TCA GGA TGC CGG CTT - 3'	53°	
		5' - CAA GGA AGA AGA ATT AGG GG - 3'		
	3' UTR-P	5' - CTC TTA AGT GCA CCT AGC T - 3'	53°	
		5' - GGT CTC TAA GCA GGA TGC TAC AG - 3'		
TGFB1	5'UTR	5' - CCA TCT CCC TCC CAC CTC - 3'	53°	94°C - 5 min.
		5' - GTC TCC CGG CAA AAG GTA G - 3'		94°C - 30 sec. } 35 cycles
	exon 1	5' - GCG CAT CCT AGA CCC TTT CT - 3'	58°	__°C - 30 sec.
		5' - CTG CCA GTC ACT TCC TAC CC - 3'		72°C - 45 sec.
	exon 2	5' - ACT CCA CCC CAG AGC TGT C - 3'	58°	72°C - 2 min.
		5' - GTA ACC AGC CGA CCC ACA -3'		4°C - hold
	exon 3	5' - AGG AAA CAG GCC TCG AGA A - 3'	58°	
		5' - AGC CAA TCA CTC AGG TTT CC - 3'		
	exon 4	5' - GAG AGA GCT GCA GTG AGA GG - 3'	56°	
		5' - AGA CAG ATG CTC AGC CCA AG - 3'		
	exon 5	5' - GGG AGG CAG GAA GGA AGC - 3'	56°	
		5' - CCA GAC CTC ATC CCC TGA G - 3'		
	exon 6	5' - TGT CCT CAG TCC ACA GTA GAT CC - 3'	55°	
		5' - TCA TAG CTC ATC TCC CTC TGG - 3'		
	exon 7	5' - CGA ATT GGA GAT GGG AAG AG - 3'	54°	
		5' - GGG CAA AGG AAT AGT GCA GA - 3'		
	3'UTR	5' - GGT CCT GGC CCT GTA CAA C - 3'	53°	
		5' - ATC CCC CAC TAA AGC AGG TT - 3'		

TGFB3	exon 1	5' - CTC TCG CAG TGC AGT GAG TT - 3'<br>5' - CTC CAG TTC AGA CCC TCC A - 3'	55°	94°C - 3 min.<br>94°C - 30 sec. }<br>__°C - 30 sec. } 35 cycles<br>72°C - 30 sec. }<br>72°C - 3 min.<br>4°C - hold
	exon 2	5' - AGC ACA CTG TTC CTG CAT - 3'<br>5' - AAG GAA CCA GCT TTC CC - 3'	55°	
	exon 3	5' - AAG CTG GTT CCT TTG CCA TA - 3'<br>5' - GGG CCT GTC TTA CAG TGA GC - 3'	55°	
	exon 4	5' - CTT CCC CAT CAC ACA GAG GT - 3'<br>5' - CCA CTC ACC CAT CCT ACC AT - 3'	55°	
	exon 5	5' - CAA GGG CTC TGC TCT CTT CA - 3'<br>5' - AGG CAG GCA GTA GAT GTT GG - 3'	55°	
	exon 6	5' - ATG GCA TGA AAG GAC TCC AA - 3'<br>5' - TGCTCACTCAACATTCAATCCT - 3'	55°	
	exon 7	5' - TGGCCTTCCAACCTCTCAC - 3'<br>5' - GCAGTGGTGGTTCTCTCTCC - 3'	58°	
