# Supplementary material for: Medical Sequencing of Candidate Genes for Nonsyndromic Cleft Lip and Palate
Source: PLoS Genet. 2005 Dec 2;1(6):e64. doi: 10.1371/journal.pgen.0010064 (PMC1298935; doi:10.1371/journal.pgen.0010064)
Supplement: Supplementary file 3 [file pgen.0010064.st002.pdf]

Exonic splicing enhancer prediction analysis for the missense mutations found in the present study (predictions below thresholds are not shown). The site of the LHX8 E221A mutation did not have any predicted exonic splicing enhancers.

Missense mutation		Motifs and (scores) by protein (threshold applied)			
		SF2/ASF (1.956)	SC35 (2.383)	SRp40 (2.67)	SRp55 (2.676)
FOXE1 A207V	Wild type	CGCACCG (2.837347)	GGCCCGCA (4.442013)	-	-
	Variant	GGCCCGT (3.217771)	GGCCCGTA (5.086453)	-	-
FOXE1 D285V	Wild type	GCCCCGA (2.292778) CCGACGG (3.890621)	GGCCCCGA (3.681759)	-	-
	Variant	-	-	-	CCCGTC (3.001157)

GLI2 R46H	Wild type	CGCAGCA (2.960014)	-	-	CGCAGC (3.882597)
	Variant	CACAGCA (3.407236)	-	GCACAGC (2.977429)	CACAGC (3.273937)
GLI2 R426Q	Wild type	CACGCGG (2.953728)	-	-	CGCGGA (4.126018)
	Variant	CGCAGAA (2.960014)	-	-	CGCAGA (3.517358)
GLI2 G778D	Wild type	TGCACGG (2.464597)	GGCCAGCG (4.419494)	-	CACGGC (3.882597)
	Variant	TGCACGA (3.191379)	GACCAGCG (4.058642)	CGACCAG (2.841356)	-
GLI2 S1213Y	Wild type	-	-	CCTCCCG (2.877836)	-
	Variant	-	-	CTACTCC	

				(4.261669)	
JAG2 M597I	Wild type	-	GATGCCTG (2.823733)	TGCCTGG (2.861579)	-
	Variant	-	GATACCTG (2.823733)	-	-
JAG2 A657H	Wild type	-	GCCTTCCG (2.781056)	-	-
	Variant	GACACCT (2.080804)	-	-	-
MSX1 P147Q	Wild type	-	CGCCGCCG (2.881025)	-	-
	Variant	CAGCCGC (3.142671)	TTCTCCCA (2.429161) AGCCGCCG (3.157467)	TCCCAGC (3.086215)	-

MSX2 S63C	Wild type	-	CGTCCCCG (3.209772)	-	GGCGTC (3.326610)
	Variant	-	GCCCGCTG (3.517899)	-	-
SATB2 T190A	Wild type	-	-	-	-
	Variant	AGCACGT (3.283441)	-	-	-
SKI A388V	Wild type	-	-	TGTCAGC (3.612945)	-
	Variant	-	TGTCAGTG (2.445360)	-	-
SPRY2 D20A	Wild type	GTGACGG (2.916245)	-	-	-
	Variant	-	TGCCGGTG (2.589028)	-	-

SPRY2 K68N	Wild type	-	-	-	-
	Variant	-	CAATCCTG (2.820769)	TCAATCC (3.361395)	-
TBX10 R354Q	Wild type	CATCCGG (2.558743)	-	-	-
	Variant	-	-	-	-
