# Supplementary material for: Medical Sequencing of Candidate Genes for Nonsyndromic Cleft Lip and Palate
Source: PLoS Genet. 2005 Dec 2;1(6):e64. doi: 10.1371/journal.pgen.0010064 (PMC1298935; doi:10.1371/journal.pgen.0010064)
Supplement: Supplementary file 4 [file pgen.0010064.st003.pdf]

Mutations screened in the CEPH Diversity Cell Line Panel.

Mutations	Findings in the CEPH Diversity Cell Line Panel
LHX8 E221A	17 samples: one Biaka Pygmy, one Chinese Han, one Chinese Miaozu, one Burusho, one Adygei, three Russian, and nine Amerindians - two Pima from Mexico, two Maya from Mexico, two Colombians, two Karatiana from Brazil, and one Surui from Brazil
MSX1 P147Q	None
SATB2 T190A	None
SKI A388V	9 samples: five Mozabite, one Palestinian, one Druze, one Cambodian, and one French Basque
SPRY2 D20A	60 samples: one Biaka Pygmy, two Bantu, five Bedoin, 13 Druze, four Palestinian, two Brahui, two Hazara, one Pathan, three Sindhi, two Makrani, three Kalash, two Burusho, two Chinese Han, one Uygur, four Sardinian, one North Italian, two Tuscan, three French Basque, two Orcadian, two Adygei, one Russian, and two Pima
TBX10 R354Q	6 samples: two Balochi, two Makrani, one Orcadian, and one Russian
