# Supplementary material for: Medical Sequencing of Candidate Genes for Nonsyndromic Cleft Lip and Palate
Source: PLoS Genet. 2005 Dec 2;1(6):e64. doi: 10.1371/journal.pgen.0010064 (PMC1298935; doi:10.1371/journal.pgen.0010064)
Supplement: Supplementary file 5 [file pgen.0010064.st004.pdf]

Markers selected for linkage disequilibrium studies.

Gene	Marker	Frequency of the rare allele	
		Filipinos	Iowa
GLI2	Snp1 = rs280199	0.379	0.244
	Snp2 = rs4848122	0.360	0.317
	Snp3 = rs7557372	0.112	0.224
	Snp4 = rs277547	0.295	0.419
JAG2	Snp1 = rs1057744	0.192	0.5
	Snp2 = rs9972231	0.206	-
	Snp3 = rs2816679	-	0.05
MSX2	Snp1 = rs4242182	0.058	0.103
	Snp2 = rs14459	0.079	0.132
SATB2	Snp1 = rs994185	0.192	0.108
	Snp2 = rs1348813	0.313	0.225
SKI	Snp1 = rs2017143	0.415	0.432
	Snp2 = rs2843159	0.145	0.137
SPRY2	Snp1 = rs504122	0.392	0.352
	Snp2 = rs4728	0.340	0.272
TBX10	Snp1 = rs2252577	0.089	0.102
	Snp2 = rs3758938	0.169	0.285
