# Supplementary material for: Medical Sequencing of Candidate Genes for Nonsyndromic Cleft Lip and Palate
Source: PLoS Genet. 2005 Dec 2;1(6):e64. doi: 10.1371/journal.pgen.0010064 (PMC1298935; doi:10.1371/journal.pgen.0010064)
Supplement: Supplementary file 6 [file pgen.0010064.st005.pdf]

Haplotype frequencies.

Gene	Haplotype (Snp1-Snp2-Snp3-Snp4 for *GLI2* and Snp1-Snp2 for all others)	Haplotype frequency	
		Filipinos	Iowa
GLI2	Common allele-common allele-common allele-common allele (Hap1)	0.236	0.255
	Rare allele-common allele-common allele-common allele (Hap2)	0.197	0.094
	Common allele-rare allele-common allele-rare allele (Hap3)	0.126	0.104
	Common allele-rare allele-common allele-common allele (Hap4)	0.114	0.086
	All other combinations	0.325	0.461
JAG2	Common allele-common allele	0.611	0.508
	Rare allele-common allele	0.185	0.439
	Rare allele-rare allele	0.197	0.052
	Common allele-rare allele	0.008	0.001
MSX2	Common allele-common allele	0.908	0.858
	Rare allele-common allele	0.012	0.023
	Rare allele-rare allele	0.044	0.069
	Common allele-rare allele	0.036	0.050

SATB2	Common allele-common allele	0.510	0.786
	Rare allele-common allele	0.176	0.015
	Rare allele-rare allele	0.013	0.089
	Common allele-rare allele	0.300	0.110
SKI	Common allele-common allele	0.559	0.562
	Rare allele-common allele	0.300	0.293
	Rare allele-rare allele	0.111	0.131
	Common allele-rare allele	0.031	0.014
SPRY2	Common allele-common allele	0.316	0.404
	Rare allele-common allele	0.344	0.316
	Rare allele-rare allele	0.062	0.006
	Common allele-rare allele	0.278	0.275
TBX10	Common allele-common allele	0.761	0.659
	Rare allele-common allele	0.079	0.053
	Rare allele-rare allele	0.010	0.040
	Common allele-rare allele	0.150	0.248
