# Supplementary material for: Medical Sequencing of Candidate Genes for Nonsyndromic Cleft Lip and Palate
Source: PLoS Genet. 2005 Dec 2;1(6):e64. doi: 10.1371/journal.pgen.0010064 (PMC1298935; doi:10.1371/journal.pgen.0010064)
Supplement: Supplementary file 7 [file pgen.0010064.st006.pdf]

Linkage disequilibrium studies of candidate genes for clefting.

Phenotype		GLI2				JAG2		MSX2		SATB2		SKI		SPRY2		TBX10	
		Snp1	Snp2	Snp3	Snp4	Snp1	Snp2/3	Snp1	Snp2	Snp1	Snp2	Snp1	Snp2	Snp1	Snp2	Snp1	Snp2
All clefts																	
Filipinos	# informative	264	272	115	250	86	68	40	65	127	150	167	111	177	158	80	75
	p-value	0.59	0.29	0.72	0.19	0.23	0.09	0.02	0.12	0.62	0.94	0.27	**0.000004**	0.89	0.51	1	0.82
Iowa	# informative	77	81	66	82	84	12	22	59	45	62	76	72	91	68	38	71
	p-value	1	0.13	0.81	0.05	0.57	0.4	0.04	0.54	0.11	0.08	0.91	0.04	0.16	0.57	0.26	0.83
Cleft lip only																	
Filipinos	# informative	64	66	27	62	34	27	11	18	44	44	51	40	60	52	25	15
	p-value	0.45	0.07	0.36	0.49	0.1	0.6	0.36	0.37	0.77	0.89	0.51	0.003	0.26	0.37	0.86	0.79
Iowa	# informative	16	22	11	21	13	1	2	9	11	11	9	7	23	18	4	12
	p-value	0.82	0.1	0.76	0.57	0.03	0.31	0.15	0.52	0.56	0.59	0.36	1	0.72	1	0.04	1
Cleft lip and palate																	
Filipinos	# informative	181	185	82	168	51	41	29	46	84	99	115	69	118	110	51	56
	p-value	0.47	0.41	0.66	0.45	0.52	0.01	0.03	0.25	0.61	0.85	0.42	**0.0002**	0.38	0.93	0.89	0.89
Iowa	# informative	36	35	30	33	26	5	6	21	15	22	26	25	28	26	12	26
	p-value	0.88	0.53	0.18	0.21	0.38	0.65	0.7	0.66	0.43	0.33	0.31	0.35	0.86	0.72	0.56	0.22
Cleft lip with or without cleft palate																	
Filipinos	# informative	264	272	115	250	84	68	40	64	126	143	164	109	177	158	75	71
	p-value	0.59	0.29	0.72	0.19	0.19	0.09	0.02	0.15	0.68	0.82	0.29	**0.000004**	0.89	0.51	0.91	0.82
Iowa	# informative	50	56	41	52	39	6	8	30	26	33	35	32	51	44	16	38
	p-value	0.9	0.17	0.3	0.39	0.05	0.41	0.31	0.47	0.33	0.27	0.66	0.41	0.71	0.78	0.61	0.31
Cleft palate only																	
Iowa	# informative	-	-	-	-	19	3	5	14	7	13	14	19	25	12	12	13
	p-value	-	-	-	-	0.04	0.31	0.17	0.63	0.09	0.31	0.31	0.12	0.055	1	0.78	0.61
Cleft lip and palate + cleft palate only																	
Iowa	# informative	36	35	30	33	45	8	11	35	22	35	40	44	53	38	24	39
	p-value	0.88	0.53	0.18	0.21	0.5	0.31	0.24	1	0.1	0.16	0.78	0.08	0.14	0.76	0.84	0.47

Bold indicates statistically significant results; blue indicates p-values that would be significant according to a less strict criteria.
