# Supplementary material for: Medical Sequencing of Candidate Genes for Nonsyndromic Cleft Lip and Palate
Source: PLoS Genet. 2005 Dec 2;1(6):e64. doi: 10.1371/journal.pgen.0010064 (PMC1298935; doi:10.1371/journal.pgen.0010064)
Supplement: Supplementary file 8 [file pgen.0010064.st007.pdf]

Haplotype analysis.

Phenotype		GLI2				JAG2				MSX2				SATB2				SKI				SPRY2				TBX10			
		Hap1	Hap2	Hap3	Hap4	C-C	R-C	R-R	C-R	C-C	R-C	R-R	C-R	C-C	R-C	R-R	C-R	C-C	R-C	R-R	C-R	C-C	R-C	R-R	C-R	C-C	R-C	R-R	C-R
All clefts																													
Filipinos	# chromosomes	165	201	63	120	105	79	9	57	65	11	31	33	175	115	19	138	155	144	75	26	160	155	34	143	114	60	7	76
	Z-value	0.76	-0.4	-0.6	-0.8	1.7	-0.5	-1.2	-1.6	2.5	-3.1	-1	-1	1.7	-0.9	0.7	-1.3	2.5	0.7	-3	-3.7	0.6	0.3	-0.7	-0.6	0.2	-0.6	-0.6	0.4
	p-value	0.44	0.61	0.52	0.41	0.07	0.55	0.22	0.09	0.009	0.001	0.29	0.31	0.08	0.36	0.44	0.16	0.01	0.42	0.002	**0.0002**	0.6	0.7	0.42	0.53	0.81	0.68	0.89	0.54
Iowa	# chromosomes	67	49	33	37	64	70	10	0	35	7	14	20	54	7	34	35	69	64	50	13	79	74	3	61	75	23	15	67
	Z-value	-2	-0	0.93	0.07	0	-0.3	0.8	-	2.1	-2.6	-0.5	-0.8	-1.6	-0.1	2.1	0.2	0.2	0.7	-0.4	-1.9	0.5	-1.1	-	0.69	0	-0.1	-1.7	0.8
	p-value	0.03	0.96	0.34	0.94	0.99	0.75	0.4	-	0.02	0.008	0.59	0.37	0.09	0.84	0.03	0.8	0.81	0.44	0.63	0.04	0.6	0.3	-	0.49	0.98	0.85	0.08	0.4
Cleft lip only																													
Filipinos	# chromosomes	40	52	13	14	41	32	2	23	18	2	9	9	48	42	7	42	49	42	29	10	53	57	9	49	31	13	0	22
	Z-value	0.74	0.36	1.19	-1.3	0.5	-1.1	-1.4	0.6	1.6	-1.4	-1.2	-0.3	0.1	-0.1	1.7	-0.4	1.2	1.2	-2.2	-1.9	0.2	0.7	-	-1.4	0.4	-0.2	-	-0.3
	p-value	0.45	0.71	0.23	0.17	0.61	0.26	0.15	0.54	0.1	0.15	0.2	0.73	0.88	0.88	0.07	0.66	0.2	0.21	0.02	0.04	0.8	0.5	-	0.13	0.62	0.78	-	0.71
Iowa	# chromosomes	16	9	4	13	10	11	1	0	5	1	3	1	10	1	3	9	7	9	6	1	18	16	1	15	10	1	1	8
	Z-value	-1.2	-	-	1.7	-1.5	1.2	1	-	1.3	-1	-0.5	-1	-1	1	0.5	0.5	0.6	-0.6	0.3	-1	0.6	-0.7	-	0	0.5	-1	-1	0
	p-value	0.22	-	-	0.07	0.13	0.22	0.31	-	0.17	0.31	0.56	0.31	0.31	0.31	0.56	0.56	0.52	0.52	0.7	0.31	0.5	0.5	-	1	0.56	0.31	0.31	1
Cleft lip and palate																													
Filipinos	# chromosomes	110	144	50	44	63	34	7	46	46	8	22	24	121	74	11	93	104	101	44	15	108	99	24	98	81	46	5	48
	Z-value	0.72	-0.2	0.22	-0.8	2.2	-2.8	-0.8	-0.2	2	-2.8	-1	-0.3	0.2	-0.5	0.5	0	2.1	0.2	-2	-3.2	0.2	0	-1.3	0.53	0	0.3	0.2	-0.3
	p-value	0.47	0.77	0.81	0.37	0.02	0.004	0.4	0.77	0.04	0.004	0.3	0.69	0.76	0.55	0.55	0.97	0.03	0.83	0.03	0.001	0.8	1	0.17	0.59	0.95	0.72	0.79	0.73
Iowa	# chromosomes	26	17	16	10	19	20	3	0	13	2	3	8	18	1	11	15	21	20	15	1	26	21	1	24	26	9	2	21
	Z-value	-1.3	0.1	-0.2	-0.1	-0.1	0	0.5	-	0.8	-1.4	0.5	-0.7	-1	-1	0.9	0.7	-1.3	2.2	-0.9	1	-0.4	0.7	-	0	-1.4	0.3	1.4	1
	p-value	0.16	0.85	0.78	0.92	0.84	1	0.56	-	0.4	0.15	0.56	0.47	0.29	0.31	0.36	0.46	0.17	0.02	0.34	0.31	0.6	0.5	-	0.99	0.14	0.73	0.15	0.31
Cleft lip with or without cleft palate																													
Filipinos	# chromosomes	165	201	63	120	103	57	9	77	65	11	31	33	167	114	18	135	152	141	73	25	159	154	34	147	111	59	6	70
	Z-value	0.76	-0.4	-0.6	-0.8	1.8	-1.6	-1.2	-0.7	2.5	-3.1	-1	-1	0.4	-0.7	1.4	-0.2	2.4	0.8	-3	-3.7	0.3	0.6	-0.7	-0.5	0.3	0.1	0.4	-0.6
	p-value	0.44	0.61	0.52	0.41	0.05	0.09	0.22	0.47	0.009	0.001	0.29	0.31	0.63	0.46	0.13	0.81	0.01	0.38	0.002	**0.0002**	0.8	0.6	0.42	0.58	0.74	0.87	0.68	0.52
Iowa	# chromosomes					29	31	4	0	18	3	11	4	28	2	20	18	28	29	21	2	44	37	2	39	36	11	6	31
	Z-value					-1	0.7	1	-	1.4	-1.7	-0.9	0	-1.4	0	1	0.8	-0.9	1.5	-0.6	0	0	0	-	0	-1	0.2	0.1	0.9
	p-value					0.28	0.44	0.31	-	0.15	0.08	0.36	1	0.14	1	0.29	0.37	0.36	0.11	0.54	1	1	1	-	0.99	0.28	0.81	0.89	0.33
Cleft palate only																													
Iowa	# chromosomes	-	-	-	-	14	16	3	0	7	0	4	5	11	3	6	7	14	16	10	3	20	20	0	10	19	7	3	15
	Z-value	-	-	-	-	1.6	-2	0.8	-	0.7	-	-1	0	-0.6	-0.3	2.2	-0.9	1.6	-0.7	-0.2	-1.5	1.3	-1.2	-	-	0.4	0	-0.5	-0.2
	p-value	-	-	-	-	0.08	0.03	0.41	-	0.47	-	0.31	1	0.5	0.76	0.02	0.33	0.1	0.46	0.79	0.13	0.2	0.2	-	-	0.68	1	0.56	0.81
Cleft lip and palate + cleft palate only																													
Iowa	# chromosomes	42	25	21	23	33	36	6	0	20	2	7	13	29	4	17	22	37	36	25	6	46	41	1	35	45	17	8	38
	Z-value	-1.8	-0.3	0.17	1.13	0.9	-1.3	1	-	1	-1.4	-0.3	-0.5	-1.2	-0.7	2	0.1	-0.2	1.2	-0.9	-0.6	0.5	-0.3	-	0	-0.9	0.3	0.1	0.6
	p-value	0.06	0.69	0.86	0.25	0.36	0.17	0.31	-	0.27	0.15	0.7	0.59	0.21	0.43	0.03	0.91	0.78	0.22	0.36	0.5	0.6	0.7	-	0.99	0.36	0.69	0.85	0.49

CC = common allele of snp1-common allele of snp2; RC = rare allele of snp1-common allele of snp2; RR = rare allele of snp1-rare allele of snp2;
CR = common allele of snp1-rare allele of snp2 (for GLI2, haplotypes are specified in Appendix 5); bold indicates statistically significant results; blue indicates p-values that would be significant according to a less strict criteria.
