# Supplementary material for: Medical Sequencing of Candidate Genes for Nonsyndromic Cleft Lip and Palate
Source: PLoS Genet. 2005 Dec 2;1(6):e64. doi: 10.1371/journal.pgen.0010064 (PMC1298935; doi:10.1371/journal.pgen.0010064)
Supplement: Supplementary file 9 [file pgen.0010064.st008.pdf]

SKI LRT results for the South American (ECLAMC) clefting samples.

Mating types: Mother-father-Child	CL	CLP	CL/P	CP	CLP+ CP	ALL
0-?-0	0	0	0	0	0	0
0-?-1	0	0	0	0	0	0
1-?-0	0	0	0	0	0	0
1-?-1	4	14	18	4	16	23
1-?-2	10	19	29	3	21	32
2-?-1	1	14	15	0	14	17
2-?-2	44	75	119	19	91	144
TOTAL	59	122	181	26	142	216
LRT statistics:						
LnL Model II	-45.808	-132.441	-182.049	-20.747	-155.791	-214.674
LnL Model I	-49.928	-132.956	-184.443	-22.076	-156.437	-216.587
χ^2, 1 d.f.	8.240	1.031	4.788	2.656	1.291	3.825
p-value	0.0041	0.3098	0.0287	0.103	0.255	0.0505

χ^2 = -2(lnL Model I – lnL Model II), i.e. likelihood ratio χ^2 comparing Models I and II
CL = cleft lip only, CLP = cleft lip with cleft palate, CL/P = cleft lip with or without cleft palate, CP = cleft palate only, CLP+CP = cleft lip with cleft palate and cleft palate only, ALL = all families.
